# Deep learning in microbiome analysis: a comprehensive review of neural network models

**DOI:** 10.3389/fmicb.2024.1516667

**Published:** 2025-01-22

**Authors:** Piotr Przymus, Krzysztof Rykaczewski, Adrián Martín-Segura, Jaak Truu, Enrique Carrillo De Santa Pau, Mikhail Kolev, Irina Naskinova, Aleksandra Gruca, Alexia Sampri, Marcus Frohme, Alina Nechyporenko

**Affiliations:** ^1^Faculty of Mathematics and Computer Science, Nicolaus Copernicus University in Toruń, Toruń, Pomeranian, Poland; ^2^Computational Biology Group, IMDEA Food Institute, Madrid, Spain; ^3^Institute of Molecular and Cell Biology, University of Tartu, Tartu, Estonia; ^4^Department of Mathematics, University of Architecture, Civil Engineering and Geodesy, Sofia, Bulgaria; ^5^Department of Applied Computer Science and Mathematical Modeling, Faculty of Mathematics and Computer Science, University of Warmia and Mazury in Olsztyn, Olsztyn, Poland; ^6^Department of Computer Networks and Systems, Silesian University of Technology, Gliwice, Poland; ^7^British Heart Foundation Cardiovascular Epidemiology Unit, Department of Public Health and Primary Care, University of Cambridge, Cambridge, United Kingdom; ^8^Victor Phillip Dahdaleh Heart and Lung Research Institute, University of Cambridge, Cambridge, United Kingdom; ^9^Molecular Biotechnology and Functional Genomics, Technical University of Applied Sciences Wildau, Wildau, Brandenburg, Germany; ^10^Department of System Engineering, Kharkiv National University of Radioelectronics, Kharkiv, Ukraine

**Keywords:** microbiome, deep learning, classification, natural language processing, clustering

## Abstract

Microbiome research, the study of microbial communities in diverse environments, has seen significant advances due to the integration of deep learning (DL) methods. These computational techniques have become essential for addressing the inherent complexity and high-dimensionality of microbiome data, which consist of different types of omics datasets. Deep learning algorithms have shown remarkable capabilities in pattern recognition, feature extraction, and predictive modeling, enabling researchers to uncover hidden relationships within microbial ecosystems. By automating the detection of functional genes, microbial interactions, and host-microbiome dynamics, DL methods offer unprecedented precision in understanding microbiome composition and its impact on health, disease, and the environment. However, despite their potential, deep learning approaches face significant challenges in microbiome research. Additionally, the biological variability in microbiome datasets requires tailored approaches to ensure robust and generalizable outcomes. As microbiome research continues to generate vast and complex datasets, addressing these challenges will be crucial for advancing microbiological insights and translating them into practical applications with DL. This review provides an overview of different deep learning models in microbiome research, discussing their strengths, practical uses, and implications for future studies. We examine how these models are being applied to solve key problems and highlight potential pathways to overcome current limitations, emphasizing the transformative impact DL could have on the field moving forward.

## Introduction

The diverse microbial communities inhabiting different environments play pivotal roles in shaping ecosystem dynamics, influencing nutrient cycling, and impacting the health and wellbeing of host organisms (Sessitsch et al., 2023; Liao et al., 2024). Understanding the intricate relationships within microbiomes is crucial for various fields such as agriculture, medicine, and environmental science. Microbiome engineering, aimed at manipulating microbial communities to achieve desired outcomes, requires comprehensive knowledge of microbial community composition, function, and interdependencies (Berruto and Demirer, 2024; Cullen et al., 2020; Lee, 2023).

Conventional analytical methods often struggle to fully capture the intricate complexity and dynamics present in microbiome data. This limitation has motivated researchers to explore advanced computational approaches such as machine learning and deep learning. Microbiome data is inherently high-dimensional, sparse, and context-dependent, posing difficulties for traditional machine learning methods. Deep learning (DL) models, with their capacity to process complex, non-linear relationships, have shown promise in overcoming these limitations. Deep learning architectures, in particular, provide robust tools for extracting meaningful patterns from complex, high-dimensional data, making them well-suited for microbiome analysis. Unfortunately, significant challenges remain. Issues such as the limited number of observations, sparse data, interpreting model outcomes, and ensuring model robustness across different types of microbiome data pose ongoing hurdles.

This paper is a complementary work and a continuation of the previous efforts carried out by the COST (European Cooperation in Science and Technology) Action CA18131 on Statistical and Machine Learning Techniques in Human Microbiome Studies (ML4Microbiome). It aims to assist microbiologists and biomedical scientists who are beginning their journey or wish to delve deeper into specialized resources that integrate machine learning techniques for the analysis of microbiome data. Previously, we described the applications of machine learning in human microbiome studies (Marcos-Zambrano et al., 2021; Moreno-Indias et al., 2021), cataloged the most common ML-based software and framework resources (Marcos-Zambrano et al., 2023) and discussed the challenges and best practices in the use of ML methods in microbiome data (Marcos-Zambrano et al., 2021; Papoutsoglou et al., 2023).

In this paper, we focus on and explore in depth the use of deep learning architectures and their applications in analyzing microbiome data, building on ML4Microbiome work where these methods were only briefly described. The rapid increase in microbiome data, driven by advances in high-throughput sequencing technologies and large-scale collaborative projects, provides a rich resource for deep learning applications. Furthermore, continuous developments in deep learning algorithms and frameworks (such as TensorFlow, PyTorch, and Keras) have made these techniques more accessible and user-friendly. New architectures and optimization techniques are being designed to address the challenges posed by high-dimensional, sparse microbiome data more effectively. These advancements collectively lower the barriers to adopting deep learning, highlighting its potential to enhance microbiome research significantly. Consequently, we anticipate a rapid increase in the use of deep learning methods in microbiome studies in the coming years. Therefore, the aim of this manuscript is to develop a more comprehensive understanding of how various deep learning architectures can improve our insights into microbiome dynamics, functions, and interactions within microbial communities and with hosts. The paper surpasses previous reviews focused on ML techniques that merely describe deep learning approaches for the analysis of microbiome datasets (Hernández Medina et al., 2022; Geman et al., 2018; Mathieu et al., 2022; LaPierre et al., 2019; Deng et al., 2021; Roy et al., 2024). It introduces non-specialized readers without background technical knowledge to a clear understanding of various deep learning architectures, along with their specific applications in microbiome analysis, illustrated by diverse examples and schemes. Additionally, the paper engages in discussions regarding their strengths, weaknesses, and challenges in the microbiome analysis.

The manuscript is structured first to highlight key applications of deep learning in microbiome research, which include data preprocessing, feature extraction and engineering techniques. This is followed by microbiome analysis tasks benefiting from deep learning approaches, such as Classification/Prediction tasks, studying microbiome interactions, clustering analysis, and using deep learning for creating metagenome-assembled genomes. Next, we describe multiple deep learning architectures following the structure of the Neural Network zoo, a comprehensive visual guide of different types of neural network architectures (Leijnen and Veen, 2020). For each architecture, we discuss its potential usefulness in the context of microbiome analysis, highlighting specific reasons. We provide a general overview of each architecture's concept and then discuss how they can be applied to microbiome-specific tasks, drawing from existing literature or proposing potential applications. In addition, for the more enthusiast readers, we provide additional bibliography that may serve as a practical guidance and to build theoretical foundations (see *Literature recommendation* in the Supplementary material 1). Finally, we discuss the risks and considerations associated with using deep learning on microbiome data. This section covers various risks, potential problems, and important considerations that researchers and practitioners should be aware of when employing deep learning techniques in microbiome research.

### Common microbiome data types

Various technologies are employed to explore the microbiome, with targeted sequencing (such as marker gene amplicon sequencing) and metagenomic shotgun sequencing standing out as two primary methods.

1. Targeted sequencing is a technique that focuses on specific regions of the genome to identify microbial communities accurately. This technique involves sequencing the amplified 16S ribosomal RNA (rRNA) gene to identify bacteria and archaea and the Internal Transcribed Spacer (ITS) region or 18S rDNA gene to identify eukaryotes. Sequencing the 16S rRNA gene is particularly important in identifying and quantifying the various bacterial and archaeal species within a sample. The analysis of the obtained sequencing data can be performed using either the Operational Taxonomic Units (OTUs) or Amplicon Sequence Variants (ASVs) approach, each providing different levels of taxonomic resolution and computational demands based on the goals of the study (Chiarello et al., 2022).
2. Metagenomic shotgun sequencing provides a more exhaustive analysis by sequencing all DNA in a sample, covering bacteria, archaea, eukaryotes, and viruses. Although this method delivers a broader overview of the microbiome, it demands more resources and computational effort. The data analysis process of shotgun sequencing data is intricate, involving the reconstruction of longer DNA sequences, taxonomic classification, and functional annotation.
3. Metatranscriptomic sequencing is an emerging technique that is used to study microbiomes. This technique involves the study of RNA transcripts to understand the active genes and the responses of the microbiome under different conditions. This approach provides valuable insights into the functional dynamics and gene expression profiles of microbial communities.
4. Metaproteomic analysis examines the proteins present in a microbiome, offering insights into the active metabolic processes within microbial communities. By identifying the proteins being produced, researchers can infer the functional capabilities of the microbiome.
5. Metabolomic analysis identifies small molecules, revealing metabolic activities within microbial communities and between the microbiome and host.

Integrating various types of microbiome data into multi-omics analysis is becoming increasingly common, which provides a comprehensive understanding of the microbiome's structure, function, and dynamics. Each data type offers unique insights, collectively enhancing our knowledge about microbial communities. In this regard, data transformation prior to applying DL is crucial for effectively handling microbiome sequencing data. They help to rectify compositional issues, reduce noise, adhere to statistical assumptions, and enable meaningful analysis and interpretation. In human microbiome studies, the most commonly used data transformation methods for both targeted sequencing and shotgun data are relative and normalization-based methods. These are followed by compositional transformations such as the centered log-ratio (CLR) and Isometric log-ratio (ILR) methods (Ibrahimi et al., 2023). Microbiome data is most often represented as a matrix or table, with each row representing a sample or subject and each column representing microbial features. However, the data can also be organized as a time series, where each time step corresponds to a different point in time (e.g., longitudinal microbiome data). In Supplementary Table 1 you can find the most common manner to feed data to the different NN architectures.

## Applications of DL techniques in microbiome research

In this section, we will explore key applications of deep learning in microbiome research, categorized into three main groups. First, we will begin by exploring DL uses for microbiome taxonomic and functional profiling (microbial taxons, derived proteins, and metabolites). Then examining data preprocessing tasks, such as data augmentation and imputation, batch correction, feature extraction, and multi-view analysis techniques relevant to microbiome data analysis. Finally, we will discuss various microbiome analysis tasks that benefit from deep learning approaches, including Classification/Prediction tasks, studying microbiome interactions and clustering analysis. In the text and Table 1, you will find a general overview of suitable architectures for each task. Architectures are selected based on known applications of the architecture for analysis of microbiome data or similar contexts. Architectures highlighted in bold indicate instances where we have found examples of their usage in microbiome data analysis in the literature. The most relevant publications were selected that showcased the versatility and effectiveness of each neural network model across different microbiome-related applications.

**Table 1:** Applications of DL techniques in microbiome research.

**Task category**	**Specific task**	**Architecture**
**Microbiome taxonomic and functional profiling**	Functional annotation and metagenome-assembled genomes (MAGs)	**FFNN**, **RNN, LSTM, Autoencoder**, **VAE**, **CNN**, **GNN, NLP**
**Data preprocessing**	Augmentation	HN, BM, CNN, **GAN**, DRN, SOM, GNN, **NLP**
	Imputation	**RNN**, HN, BM, **GAN**
	Batch correction	**Autoencoder, GAN**
	Feature extraction and engineering	**Autoencoder**, **VAE**, **CNN**, **Attention N**, **GNN**, **NLP**
**General applications**	Classification/Prediction	**FFNN**, **RNN**, **LSTM**, **Autoencoder**, **VAE**, **BM**, **CNN**, **GNN**
	Microbiome interactions	**LSTM**, **Autoencoder**, **VAE**, **HN**, **BM**, **CNN**, **DRN**, **Attention N**
	Clustering	**Autoencoder**, VAE, **SOM**
	Multi-view analysis	**LSTM**, Autoencoder, VAE, **CNN**, **NLP**

Specific tasks and models described in the literature (bold) or suitable (regular text) for that task are shown.

### Microbiome taxonomic and functional profiling

The identification of microbiome features (i.e., taxa, genes) is essential for posterior functional studies and profiling of ecosystems that could be done in a metagenomic project. Numerous tools had previously been developed for these tasks (reviewed in Marcos-Zambrano et al., 2023). The spread of the shotgun sequencing method has led to the study of the functional microbiome, allowing for the characterization of microbiome small molecules (toxins, antibiotics, etc.) and their functionality (Zhang Y. et al., 2022; Ma et al., 2022). The initial step involves identifying these molecules which typically are encoded in biosynthetic gene clusters (BGCs). To facilitate this, different models were developed, including pHMM, BLAST, and ClusterFinder (reviewed in Ak and Sy, 2018). DL has enhanced the accuracy of these algorithms while also delivering good computational performance for some of them. The emergence of deep learning models has led to the development of new models for this purpose, such as e-DeepBGC (Liu M. et al., 2022) or DeepRFI (Gligorijević et al., 2021). Another emerging aspect of microbiome taxonomic and functional profiling is the creation of metagenome-assembled genomes (MAG). The approach is based on the reduction of reads to smaller contiguous sequences (contigs) with significant overlap and binning them, i.e., grouping them by their genome of origin. The process of binning is a complicated process that typically relies on the analysis of the detected sequences' co-abundance (contigs from the same organism should have abundance's high covariance across samples) or the k-mer frequency found in the DNA. There are three main groups of binning approaches based on the features utilized. These groups include sequence composition (k-mer frequency) based, abundance (contig coverage) based, and hybrid methods (combining both k-mer frequency and coverage features). However, using these feature sets independently can generate problems like sequence redundancy, and co-abundance trends to cause chimeric MAGs. The emergence of deep learning-based binning methods has improved the handling of heterogeneous information in the process of MAG recovery.

### Data preprocessing

#### Augmentation

Microbiome data poses a significant challenge due to their high dispersion and sparsity, requiring a substantial amount of data to build statistical models effectively. However, not all microbiome studies have the resources to collect large datasets. Consequently, creating augmented datasets to train more sophisticated statistical models has become a viable approach in the microbiome field. These generated datasets exhibit similar characteristics to real microbiome data, preserving the sparsity and diversity of the microbiome, while retaining important taxa-taxa correlations (Liu M. et al., 2022; Gligorijević et al., 2021).

#### Imputation

Data imputation is an additional method used to generate microbiome data. The microbiome is a dynamic component of organisms that evolves over time and in response to various external conditions. Therefore, longitudinal studies conducted over time or under different health conditions/treatments are precious by providing insights into the microbiome's adaptation and its impact on host health. However, these studies complicate the collection of comprehensive and complete datasets due to the need for data from different time points, adding to the intrinsic complexity of microbiome data mentioned earlier. Missing data at specific intervals is a common challenge, potentially hindering the development of robust statistical models. To address this, DL techniques have also been employed to impute these missing points (Choi et al., 2023), aiding in completing the datasets necessary for the successful development of ML models.

#### Batch correction

Combining various microbiome studies is a common approach to tackle the lack of large datasets and data sparsity, effectively enlarging the pool of samples. However, integrating databases coming from different sources can be a challenging task. The batch effect, alterations in data caused by external non-biological factors in the experiment, can affect the generation of ML models. Li et al. (2023) designed a DL-based algorithm based on GAN networks for this purpose. Their algorithm, coupled with a mathematical index to predict health status (GMHI), was able to remove the batch effect while keeping the particularities of the different disease status in several studies, improving the disease discrimination in those datasets. Additionally, autoencoder-based methods can also be used for batch correction (Bank et al., 2023). They can effectively remove batch effects by compressing data and applying guided training to keep the biological variations, similar to the adversarial approach employed by GAN networks. For instance, Autoencoder-based Batch Correction (ABC) is a semi-supervised deep learning architecture designed for integrating single-cell sequencing data from multiple sources. This method removes batch effects while maintaining the biological variations in the data (Danino et al., 2024). Although designed for other purposes, this tool has a great potential use in the microbiome context.

#### Feature extraction and engineering

Feature extraction involves identifying, selecting, or creating meaningful data attributes from raw datasets to enhance model accuracy by capturing relevant information and patterns. Deep learning may be used as it is able to manage complex datasets and interpret non-linear patterns effectively. For example, this could involve quantifying specific bacterial groups or extracting pathways related to host-microbe interactions, simplifying data complexity, and improving predictive capabilities for disease states or ecosystem dynamics (Oh and Zhang, 2020; Shen Y. et al., 2023; Tataru et al., 2022).

### General applications

In previous sections, we have primarily concentrated on preprocessing data (imputation, data generation) and identifying unique features that reveal patterns. However, the main use of deep learning with microbiome data is classifying original samples into groups or populations using various types of neural networks.

#### Classification/prediction

Classification and prediction are two fundamental aspects of machine learning, each serving a unique purpose in data analysis and decision-making processes. Classification involves categorizing data into predefined groups or classes based on their features; it's primarily used when the outputs are categorical, such as diagnosing diseases (healthy vs. diseased) or identifying customer sentiment (positive, negative, or neutral). On the other hand, prediction refers to forecasting continuous outcomes based on input variables, such as blood glucose levels for Type 2 diabetes (T2D). This process, often called regression in statistical contexts, uses different methodologies like linear regression or deep learning models to estimate numerical values.

#### Microbiome interactions

The primary use of deep learning is to predict health or disease states based on microbiome data. Determining whether a particular microbiome is linked to disease development is crucial. However, some approaches focus solely on factors influencing the microbiome's health or disease state without considering microbial interactions or environmental influences that could drive the final outcome. Models like the generalized Lotka-Volterra (gLV) have been used to understand microbial community interactions and how small changes can impact the entire community (van den Berg et al., 2022). The gLV model estimates bacteria growth rates and interactions among community members. However, it struggles with large, complex interactions, often requiring longer computational time compared to newer DL-based models.

#### Clustering

Clustering is a type of unsupervised learning technique used in data analysis where data points are grouped into groups (clusters) based on their similarities, with the aim that items in the same cluster are more similar to each other than to those in other clusters. This method is widely used across various fields, e.g., to identify inherent structures or patterns in data without prior labeling of the points. For example, clustering can be applied to patient data to identify subgroups that share similar microbiome profiles (de Kok et al., 2024), which can help tailor specific treatments or better understand the progression of diseases. Another typical example is when researchers use clustering to analyze grouping organisms or genes based on genetic similarity, which can reveal evolutionary relationships or functional similarities (Nissen et al., 2021).

#### Multi-view analysis

Recently, studying microbiomes using a combination of different omic approaches has become increasingly common. These multi-omics datasets, alone or together with host-specific data or environmental data, can be processed with multi-view analysis methods (also referred as data integration), allowing for a comprehensive understanding of the microbiome's structure, function, and dynamics. Multi-omics multi-view analysis methods have been categorized into five distinct strategies: early, mixed, intermediate, late, and hierarchical (Picard et al., 2021) and general aspects of deep learning-based multi-omics data integration methods have been reviewed by Kang et al. (2022). Early fusion involves transforming all datasets into a unified representation, which is then used as input for a chosen deep learning model. In the case of late fusion, first-level models are developed from individual data types, and then the predictions from these models are combined by training a second-level model, which serves as the final predictor. Multi-view analysis using deep learning has been explored in several microbiome studies to harness the strengths of different data types and enhance our understanding of microbial communities and their interactions.

## Deep learning architectures

In this section, we explore various deep learning architectures within the realm of microbiome analysis. We begin with a general overview of each architecture's concept before delving into its specific applications in microbiome analysis. By synthesizing insights from existing literature (see exact examples of architectures in Supplementary material 2) and proposing potential applications, our goal is to offer valuable perspectives on leveraging these architectures to overcome challenges and foster advancements in microbiome research.

**Figure 1:**
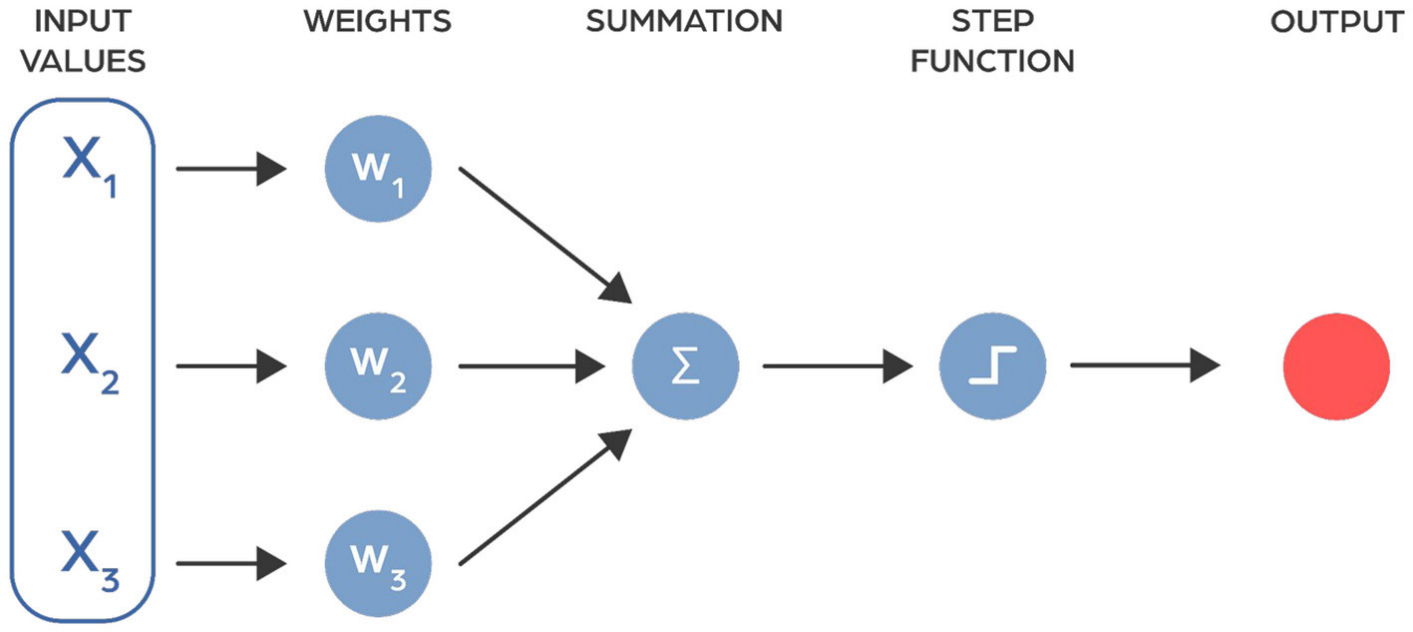
The model of neuron as proposed in 1943 by McCulloch and Pitts (1943).

Artificial neural networks are computer models inspired by the workings of the human brain. They consist of multiple layers, each containing units called neurons that process information. These neurons are connected by activation functions, enabling the network to learn and make decisions (Figure 1) (McCulloch and Pitts, 1943).

There are usually three types of layers: input, hidden, and output. The input layer receives data, with each neuron representing an element like a pixel in an image or a word in a sentence. Hidden layers, positioned between the input and output layers, process and transform this data to learn complex relationships. The output layer generates the final prediction or result, for example, identifying healthy individuals and those with specific diseases based on their gut microbiome profiles. Between layers are activation functions, which are mathematical functions used in neurons that decide whether a given neuron should be “activated,” meaning it passes the signal further. They introduce non-linearity, which allows the neural network to learn complex patterns. Examples of activation functions include ReLU (Rectified Linear Unit), which passes positive input values and returns zero for negative ones, and the sigmoid function, which transforms the input value into a range from 0 to 1, useful when predicting probabilities. You can find a summary of the most commonly used activation functions in Supplementary Table 2.

There are different mathematical metrics to measure the performance of a neural network model. The use of one or the other depends on the classification performed by the model, although some of them can be used for the same task. For example, precision and recall are metrics more commonly used for Classification/predictions of categorical classes while Mean squared error (MSE) or Root mean squared error (RMSE) are more commonly used in regression problems. See Supplementary Table 3 for a summary of the most typical evaluation metrics in DL.

### Feed forward neural networks in multi-layer perceptron type

**Figure 2:**
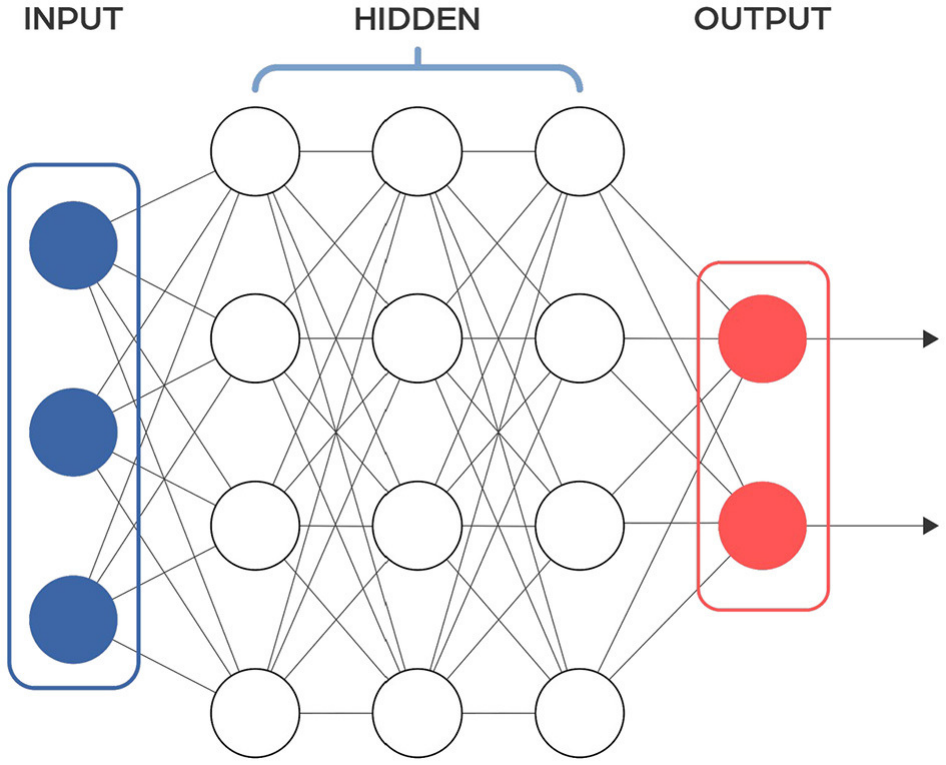
Scheme of a typical feedforward neural network architecture. Input layer receives the input data, hidden layer consists of neurons that apply a weighted sum of inputs followed by an activation function to learn complex patterns, output layer provides the final output of the network. Information flows in one direction, from the input layer, through the hidden layers, to the output layer.

Feedforward neural networks (FFNNs) are a type of neural network that passes information from input to output without looping back at any point (Figure 2). A notable subclass of FFNNs is the multilayer neural network, also known as Multilayer Perceptrons (MLPs or MLPNNs) (Rumelhart et al., 1986), which are made up of layers. Each layer connects only to the next layer in line, without any connections within the same layer. The training of MLPs employs the backpropagation algorithm within a supervised learning framework, where they learn from sets of known input-output pairs and measure their accuracy using metrics like mean squared error (MSE). Although they theoretically can model any relationship between inputs and outputs with enough neurons in the hidden layers (see Cybenko Theorem), their effectiveness in practical applications can vary. To improve their performance, FFNNs are often used together with other types of neural networks. The input to the FFNN is a finite-dimensional vector of a fixed length, which is derived from raw data through appropriate processing.

#### Functional annotation and metagenome-assembled genomes

FFNNs have been used for gene identification, using reference databases as a guide [e.g., NCBI Refseq, CARD (Jia et al., 2017), ARDB (Liu and Pop, 2009), or UNIPROT (Apweiler et al., 2004)], to improve gene identification and find new sequences (e.g., identifying new antibiotic resistance genes). That is the case of tools like Meta-MFDL (Zhang et al., 2017), Deep-ARG (Arango-Argoty et al., 2018), or ONN4MST (Zha et al., 2022). Regarding MAG generation, SemiBin (Pan et al., 2022) and SemiBin2 (Pan et al., 2023) are advanced binning algorithms that use DL. They work by dividing long contigs into two equal-length segments to create pairwise must-link constraints, and use taxonomic annotation information to create pairwise cannot-link constraints. SemiBin employs a semi-supervised autoencoder to extract this constraint information and generate embeddings for clustering. SemiBin2 is an upgraded version of SemiBin, which generates must-link constraints similarly, but introduces cannot-link constraints by randomly sampling pairs of contigs. COMEBin (COntrastive Multi-viEw representation learning for effective Binning of metagenomic contigs) is a binning method based on contrastive multi-view representation learning (Wang et al., 2024). COMEBin utilizes data augmentation to generate multiple fragments (views) of each contig and obtains high-quality embeddings of heterogeneous features (sequence coverage and k-mer distribution) through contrastive learning. The network structure used consists of two primary modules. The first module uses a FFNN to process contig coverage features. The second module also uses a FFNN to integrate the output of the first module and the k-mer features, generating an embedded representation of both. These embeddings are further used in the clustering process.

#### Classification/prediction

FFNNs can be applied to analyze microbiome data and make predictions or classifications based on the input features, data representation, feature engineering, network architecture, training and validation, evaluation, and prediction. Some of the designs used taxa abundances as input for the networks (Galkin et al., 2020; Wu et al., 2024). Others used different approaches like feeding directly k-mer distributions (Asgari et al., 2018), or combining different sources of data like taxa, metabolic and genomic abundances (Lee and Rho, 2022).

FFNNs have also been used to predict microbial community composition based on microbiome-environment interactions., MetaMLAnn algorithm tries to infer microbial communities in unsampled city areas based on the composition of sampled locations (Zhou et al., 2019).

#### Multi-view analysis

MDL4Microbiome integrates three distinct features of the microbiome: conventional taxonomic profiles, genome-level relative abundance, and metabolic functional characteristics, to improve classification accuracy (Lee and Rho, 2022). Each feature is processed through a separate supervised MLPNN. The final hidden layer of each model generates embedded representations of the respective feature. By combining these representations, a new shared representation is created that retains the essential characteristics of each of the different modalities.

### Recurrent neural networks

**Figure 3:**
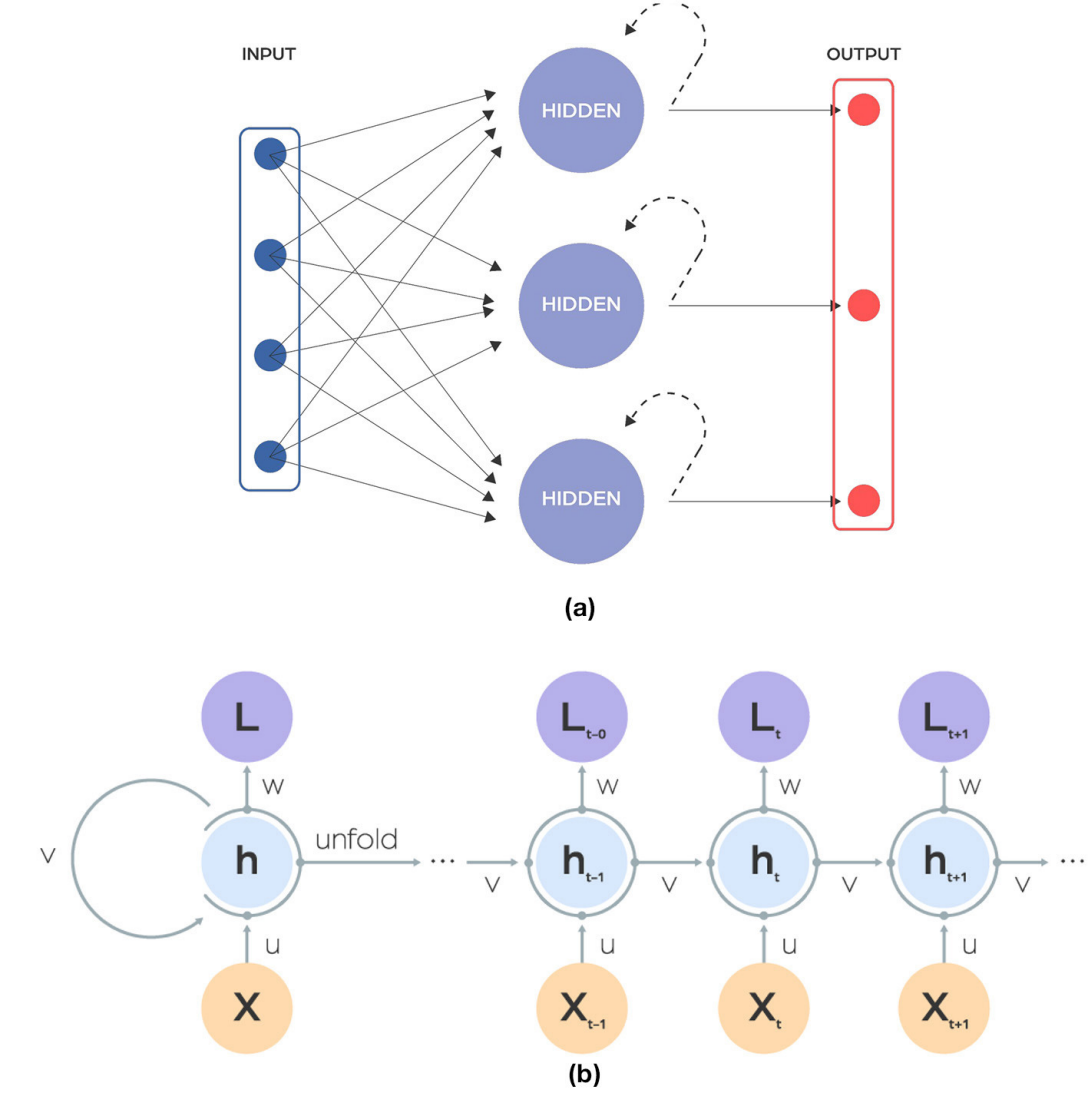
**(A)** Scheme showing a recurrent neural network (RNN) architecture. The input is a sequence of finite-dimensional vectors, each of fixed length, which are derived from raw data through appropriate processing. This type of architecture uses recurrent units in hidden layer. **(B)** Depicts the structure of the hidden layer: a single recurrent neuron (cell).

Recurrent neural networks (RNNs) are a type of neural network that adds a time dimension to data processing (Figure 3). They can remember information from previous inputs because they connect across different time steps. This ability makes them effective for tasks that rely on past information, such as predicting the next word in a sentence. However, RNNs are particularly susceptible to the common neural network issues of vanishing and exploding gradients, wherein the gradient either diminishes or increases exponentially across time steps due to the characteristics of activation functions. This phenomenon can lead to substantial information loss during training. In the field of microbiology, RNNs and LSTMs are useful for studying the dynamics of microbial communities over time. They have been used to predict changes in the composition of microbiomes, forecast how populations of microbes change, and understand how microbes interact with their hosts over time.

#### Imputation

According to Choi et al. (2023) the specific attributes of RNN architecture render it suitable for adaptation in tasks such as missing data imputation in longitudinal studies, where occasional data points are absent.

#### Classification/prediction

RNNs handle sequences incrementally, retaining a memory of past inputs via hidden states, which is advantageous for classification tasks requiring analysis of variable-length sequential data and capturing temporal dependencies (Ditzler et al., 2015).

### Long short-term memory

**Figure 4:**
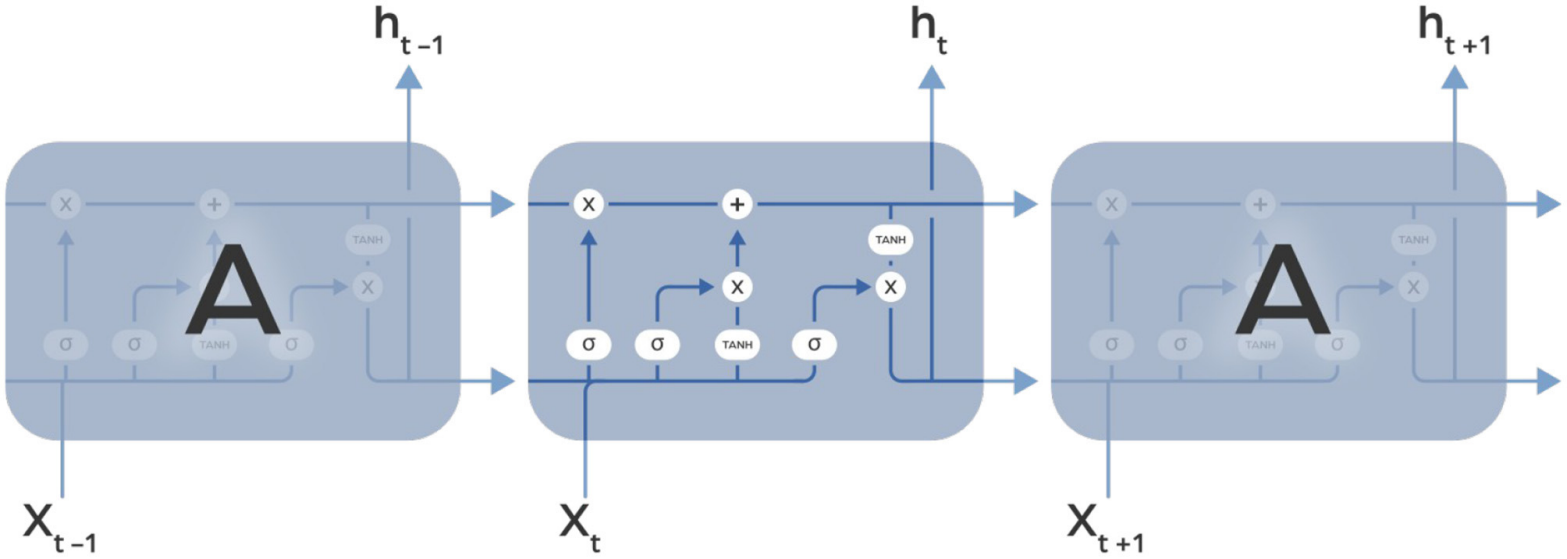
Image illustrating the unfolding of Long Short-Term Memory (LSTM) recurrent neural layer over time. The LSTM cell, highlighted in the middle, contains three gates: input, forget, and output. These gates regulate the flow of information, enabling the cell to maintain and update its state over time. The unfolding shows how the LSTM cell is reused at each time step, effectively capturing long-term dependencies in sequential data.

Long Short-Term Memory (LSTM) networks are specialized Recurrent Neural Networks designed to solve problems with vanishing and exploding gradients using a system of gates and a memory cell (Figure 4). This system, more reminiscent of an electrical circuit than biological structures, includes three gates: input, output, and forget. The input gate decides how much of the previous information to keep, the output gate controls what the next layer should know about the current state, and the forget gate lets the network ignore unnecessary information, like irrelevant details, when learning something new. LSTMs are trained with sequences of labeled data and are widely used in tasks that require an understanding of how things change over time. They are particularly good at handling data where the timing of events matters, such as analyzing temporal changes in microbiome data. Similarly, as in RNN, the input to LSTM is a sequence of finite-dimensional vectors, each of fixed length, which are derived from raw data through appropriate processing.

#### Classification/prediction

This type of network can be applied to predict disease progression or treatment outcomes based on longitudinal microbiome data. They have also been utilized in time-series classification tasks, such as identifying disease onset or detecting changes in microbial composition associated with environmental factors. The work of Metwally et al. (2019), where they used a LSTM to predict child allergies in a longitudinal study, illustrates well the potential of this architecture in this regard.

#### Microbiome interactions

Baranwal et al. (2022) proposed the use of neural networks as an alternative method to gLV. They designed an architecture based on LSTM, and trained it on microbe-microbe and microbe-metabolite interactions. The model proved to be powerful to understand those interactions, identifying important species that could be affecting the microbial community dynamics and their metabolites profile. For example, they found that certain phyla are more involved in shaping metabolite production (e.g., *Firmicutes*) while others influence community interactions more (e.g., *Bacteroides*). This research opens the possibility to shape those community relations to obtain or affect a patient's metabolic profile and thus his/her health status.

### Autoencoders and variational autoencoders

**Figure 5:**
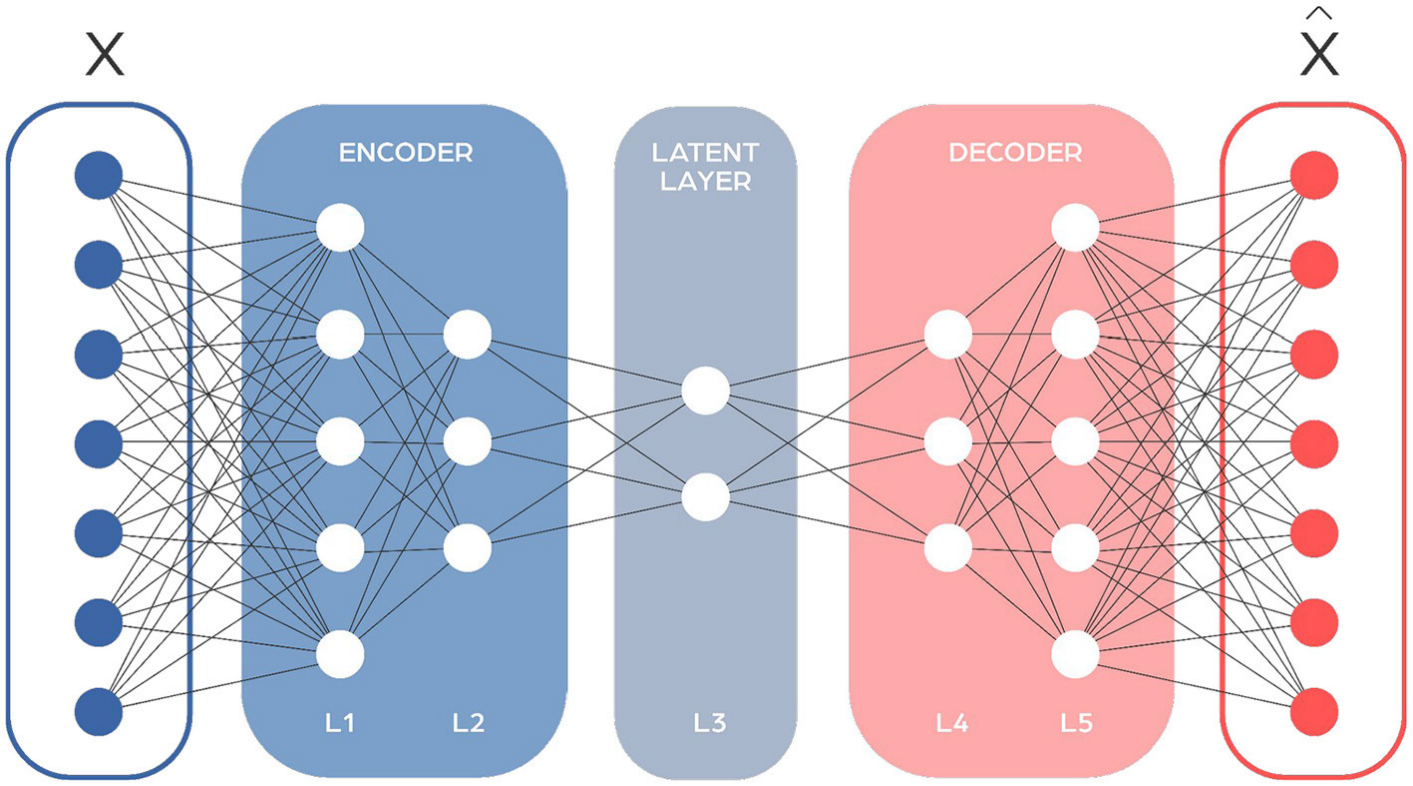
Autoencoder network architecture consists of two main components: the encoder, which processes the input sequence and compresses it into a fixed-size context vector, and the decoder, which generates the output sequence from this context vector. The input to the autoencoder is the raw data that you want to encode and compress, and the output is the reconstructed version of that data, emerging from the decoder. The effectiveness of an autoencoder is generally evaluated based on how accurately this output matches the original input.

Autoencoders (AEs) are a type of neural network used mainly for compressing information (Figure 5). They have a distinctive hourglass shape, with the narrowest section in the middle acting as the point of maximum compression. This middle point divides the network into two sections: an encoder that compresses the data, and a decoder that reconstructs it. They're designed to minimize the difference between the input and the output through backpropagation. Autoencoders can often have symmetrical designs, which means the way they compress data mirrors the way they decompress it. In microbiome research, autoencoders help in simplifying complex data by reducing its dimensionality and highlighting important features. This makes them great for tasks like spotting outliers or transferring knowledge between different studies.

Variational Autoencoders (VAEs) take autoencoders further by using a probabilistic approach. Unlike regular autoencoders that compress data to a fixed point, VAEs compress data into a range of possible values, making them good at generating new, realistic data samples. They adjust not only for the accuracy of data reconstruction but also for the realism of the compression, which is compared to a standard model, usually Gaussian. This makes VAEs powerful tools for generating varied and realistic data in complex areas like microbiome research, helping scientists understand and simulate microbial ecosystems better.

#### Functional annotation and metagenome-assembled genomes

For MAGs generation, normally contigs are generated using non-DL software designed for this purpose (reviewed in Marcos-Zambrano et al., 2023), then these contigs are passed through a DL architecture for binning and classification. Nissen et al. (2021) developed a DL-based tool, VAMB (variational autoencoder for metagenomic binning), that using autoencoders (VAE), combined both parameters (co-abundance and k-mer pattern) to implement the identification of contigs belonging to particular microbial population. Tetranucleotide frequencies (TNF) and abundances were encoded in the VAE to generate a latent layer that was later decoded into output TNF and abundance vectors. The NN integrated well the two data sources, clustering better than the two independent datasets and having a greater percentage of reconstruction increase with respect to other models like Canopy, MetaBAT2 or MaxBin2 (reviewed in Marcos-Zambrano et al., 2023; Roy et al., 2024).

CLMB (Deep Contrastive Learning for Robust Metagenomic Binning) (Zhang P. et al., 2022) is an extension of VAMB, which employs contrastive learning. Contrastive learning is a self-supervised technique that helps learn valuable representations of input data by bringing similar instances close while pushing dissimilar ones away. CLMB adds a pair of augmented data to each contig by introducing noise to the feature vector. This way, it obtains integrated representations that combine heterogeneous features. AAMB (Líndez et al., 2023), another VAMB extension, is based on adversarial autoencoders. It encodes contigs into 2 latent spaces (categorical and continuous) and then discriminates them keeping similar distance to the original distribution. Other VAMB extensions include CCVAE (Lamurias et al., 2023), introducing graphs as representations of contigs (nodes) and k/mers (edges) to constrain the autoencoding.

Other approaches combined autoencoders with non-deep learning clustering algorithms like DBSCAN for further taxa classification (Wijegunarathna et al., 2021), or with other deep learning architectures (e.g., Adversarial Deep Embedded Clustering, also based on autoencoders) to perform the binning (Bao et al., 2022).

#### Feature extraction and engineering

Autoencoders are often used to reduce dimensionality from the microbiome profile, generating a low-dimensional representation. Thus, noise and unnecessary information are filtered, and data can be easily processed to build classification models. Different groups tested alternative approaches in metagenomics, coupling feature extraction using autoencoders with machine learning algorithms as final classifiers like RF (Oh and Zhang, 2020; Shen W. X. et al., 2023; Wang et al., 2023), SVM (Oh and Zhang, 2020), gradient boosting (Shen W. X. et al., 2023), or other DL architectures like FFNN (Oh and Zhang, 2020).

#### Classification/prediction

Grazioli et al. (2022) designed a multimodal deep learning approach where data that comes from the same metagenome but with entirely different information (phylogenetic abundance, gene markers, and metabolomics) is integrated using multimodal variational information bottlenecks (MVIB). This deep network can encode the information coming from different sources, keeping the maximum information possible. This model could beat or at least match any of the previously mentioned models in various datasets, requiring less hyperparameter tuning and facilitating the interpretability of the results by revealing potential disease markers in the input data.

#### Microbiome interactions

García-Jiménez et al. (2020) used autoencoders to extract latent spaces from OTU relative abundance and environmental data, and trained this network to infer microbial community composition directly from the environmental data. The advantage of this strategy resides in its capability to make predictions of microbial composition without having to sequence samples and avoiding all the processing of this complex data.

#### Clustering

The combination of autoencoders with clustering techniques leads to methods like Deep Embedded Clustering (DEC) (de Kok et al., 2024). DEC starts by compressing the data using an autoencoder, then improves the grouping of the data by refining how it's clustered. This approach is especially good at revealing hidden patterns in microbiome data.

### Hopfield networks and boltzmann machines

Hopfield networks (HNs) (Hopfield, 1982) are unique neural networks where each neuron can act as an input, hidden, or output node at different times. Training of these networks involves setting neuron states to represent specific patterns. Then, the connections, or weights, between neurons are calculated and fixed. The network adjusts its neurons to reduce the global energy function. This process results in the formation of associative memory, as the network stabilizes into states similar to the input patterns. Each neuron in a Hopfield network can be in one of two states (spins), either −1 or 1, and the neurons can update their states all at once or one at a time using a method known as Glauber dynamics. The network stabilizes when no neuron changes its state anymore, which helps it remember patterns similar to those it learned.

Boltzmann machines (BMs) (Hinton and Sejnowski, 1983) are similar to Hopfield networks but make a clear distinction between input and hidden neurons. They start with random weights and learn by either traditional methods like back-propagation or by a special method called contrastive divergence, which adjusts weights based on a learning process. Neurons in Boltzmann machines switch between two states, influenced by a setting called “temperature.” Lowering this temperature gradually helps the network stabilize its neuron states, allowing it to settle into a balance.

#### Augmentation and imputation

HNs are associative memory tools, enabling pattern recognition and the imputation of missing data by converging to learned patterns. Similarly, BMs, as stochastic neural networks, capture complex microbial feature interactions through unsupervised learning, uncovering hidden associations and statistical properties. Despite their theoretical utility for augmentation and imputation, no specific examples of their application in microbiome contexts were found.

#### Microbiome interactions

Sokolovska et al. (2019) proposed the use of DRBM in combination with causal inference models to address the interactions between very different data sources like microbiome and health/nutritional data (glucose homeostasis marker, physical activity, etc.), to assess how the environment, like nutrients in our diet, influences microbiota dynamics. The authors combined the causal inference algorithms with Principal Component Analysis and the DRBM to generate an efficient interaction model between those parameters that is relatively simple and does not require intensive hyperparameter tuning. In other papers, they applied this model to a different problem, detecting the effect of a common drug like metformin in the human gut microbiome and improving the accuracy obtained by other methods with their algorithm's architecture (Sokolovska et al., 2020).

### Convolutional neural networks

**Figure 6:**
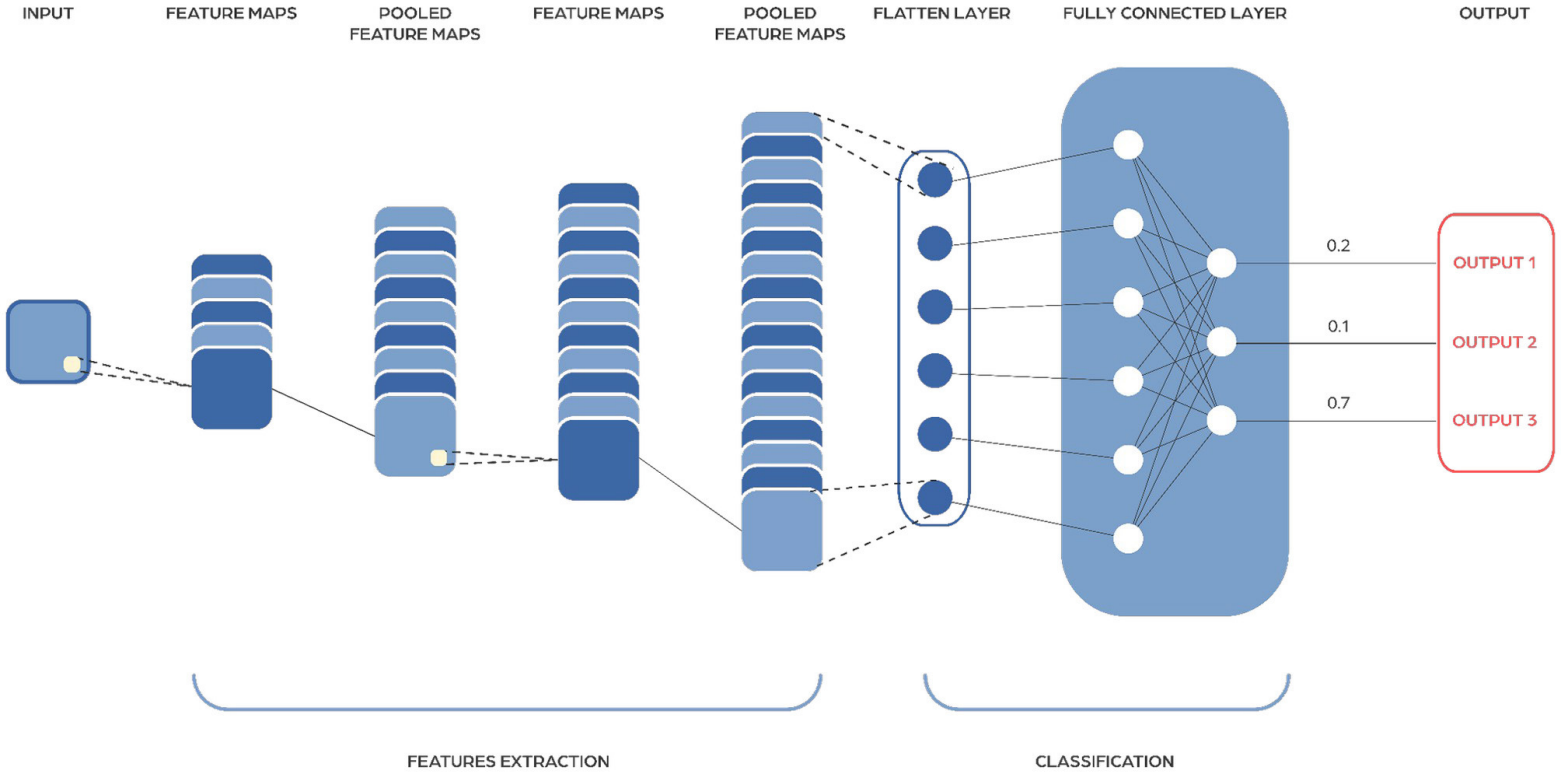
Image showing a Convolutional Neural Network architecture. They consist of multiple layers, including convolutional layers that apply filters to extract features, pooling layers that reduce dimensionality, and fully connected layers that perform classification based on the extracted features.

Convolutional Neural Networks (CNNs) work by using special layers called convolutional layers that help them extract important features from input data (Figure 6). These layers have trainable filters or kernels that move across the input, identifying patterns like edges in early stages, and more complex features deeper in the network. CNNs also use pooling layers to simplify the information by making it smaller and more manageable, while still keeping the important parts. This helps reduce the amount of work needed and speeds up processing. CNNs include non-linear activation functions, like ReLU (Rectified Linear Unit), to help them handle complex patterns, not just straight lines. Typically, CNNs end with fully connected layers, which learn to make final decisions for tasks like recognizing images or identifying objects.

The input to a CNN is generally an image or an array of images (tensor), and the output depends on the specific task: it could be categorical class probabilities for classification, pixel-wise annotations for segmentation etc. In the case of microbiome data a special transformation is required to convert the data into a CNN-friendly format.

In microbiome analysis, CNNs prove highly effective for analyzing and categorizing microbial communities. They excel in tasks such as microbial community classification, microbial localization, predicting disease risks, analyzing microbiome images, facilitating drug discovery, and conducting metagenomics studies. Researchers have started using CNNs to sort through metagenomic data, which includes all the genetic material in an environmental sample, because of their ability to handle data that has a spatial layout, much like images. CNNs are helpful especially when bacterial community composition or other microbial data types are arranged in ways resembling pictures, using presence-absence matrices or phylogenetic trees. CNNs are good at finding patterns and relationships in this kind of data, making them useful for grouping similar microbial communities together.

#### Functional annotation and metagenome-assembled genomes

The architecture most commonly used for this task and with the better outcomes has been the CNN. Several tools have been designed with this architecture to identify genes from metagenomes like CNN-MGP (Al-Ajlan and El Allali, 2019), or differentiate viral sequences in the metagenome (Fang et al., 2019, 2020; Arisdakessian et al., 2021; Ren et al., 2020; Chu et al., 2022). Other architectures developed to identify viral sequences have been RNN (Liu et al., 2022), although CNNs outperform it for this task. CNNs have also been used to determine a metagenome profile, identifying the taxa present in a certain subject. These techniques are usually based on the emergent DL-based binning methods that are trying to improve the handling of heterogeneous information in the process of genome classification using reads directly or MAG recovery. Some of the approaches developed aim first to encode through different models the genome information and then proceed to genome classification. For example, using CNNs (i.e., CNN-RAI or DeepMAsED) (Karagöz and Nalbantoglu, 2021; Mineeva et al., 2020) to encode the information from sequence co-abundances, using relative abundance index or one-hot encoding, and then using other architectures (e.g., FFNNs) (Busia et al., 2019) for classification. CNNs (Fiannaca et al., 2018) have also been applied to k-mers for encoding. In addition, other models have combined CNNs (Borgman et al., 2022) with traditional cluster algorithms like Nearest-Neighbor instead of performing a classification with other NN. Or CNN with LSTM (Liang and Sakakibara, 2021), to resolve the partitioning of a de Brujin graph at contigs chimeric nodes, generating longer contigs, reduced chimeric assembly and improving MAG resolution.

#### Features extraction and engineering

Sharma and Xu (2021) implemented CNN for feature extraction, using as input taxonomic information as an OTU vector (further described in Multi architecture designs).

#### Classification/prediction

Reiman et al. (2017) pioneered CNNs for constructing phylogenetic trees of analyzed samples, using abundance data. Although this method didn't outperform FFNNs, it advanced CNNs for clearer neural network decision-making. Reiman and others further developed CNN applications in metagenomics by adjusting data imputation, adding feature extraction for better interpretability (Reiman et al., 2020). Li et al. (2021) kept exploring this line of work, adding more information of the phylogenetic tree like the number of child nodes, nodes' distance or height of layers. Fioravanti et al. (2018) coupled an OTU distance matrix based on patristic distance (distance between two taxa) with *k* nearest neighbors computation to generate the input for the CNN. Wang et al. (2021) used patristic distance in their correlation model to cluster taxa, which became the input for their CNN. Their model outperformed other CNNs and machine learning methods like RF in prediction tasks by optimizing for dense and large clusters, even with decreasing cluster size and density. They claimed their algorithm achieved higher performance with lower computational requirements, especially effective with limited sample sizes. Chen et al. (2022) applied CNNs to shotgun metagenomic data, utilizing a pre-designed CNN for classification and subsequently extracting information from the CNN's outputs using a weighted RF. Pfeil et al. (2023) generated a radial heatmap image to provide the CNN with the OTU abundance data and retrained a publicly available CNN architecture (ResNet50) to classify the microbiome data into healthy samples and samples with T2D. Sharma et al. (2020) used CNNs and explored two distance metrics for clustering the OTUs. They clustered the OTUs by *phylum* and then ordered them based on Euclidean distance to the cluster center or correlation between bacteria. Although they achieved good results, improving the outcome of other methods in the same datasets, different limitations like the number of OTUs associated with disease considered or the correlation made only inside a *phylum* but not considering potential correlations of bacteria between different *phyla*, could have been addressed. Nguyen et al. (2018) used metagenomics relative abundance to generate 2D images that were later fed to a CNN. Deepening into this strategy, Shen W. X. et al. (2023) combined UMAP embedding and hierarchical clustering methods, taxonomically truncated, on metagenomic data in the form of a correlation matrix. This generates a multichannel image, each channel representing a taxonomic level with a variable number of clusters embedded in 2D maps and filled with abundance values. The images are provided to a CNN network that will leverage all the noise-cleaned and highly processed information to classify patients. Finally, Rahman and Rangwala (2020) applied CNN on metagenome sequences using a multiple instance learning paradigm (MIL), where individual instances (e.g., sequences) are grouped together in a bigger instance or “bag” used later on the network. In their case, they clustered sequences by k-means and created an instance (embedding) for each cluster. The embeddings were then analyzed by a CNN that determines which embeddings determine a disease state.

#### Microbiome interactions

CNNs can also be employed to predict microbial community composition of one anatomical site based on the composition of another site. In their work, Rampelli et al. (2021) designed a CNN that leveraged oral microbial composition to predict the fecal microbiome.

### Generative adversarial networks

**Figure 7:**
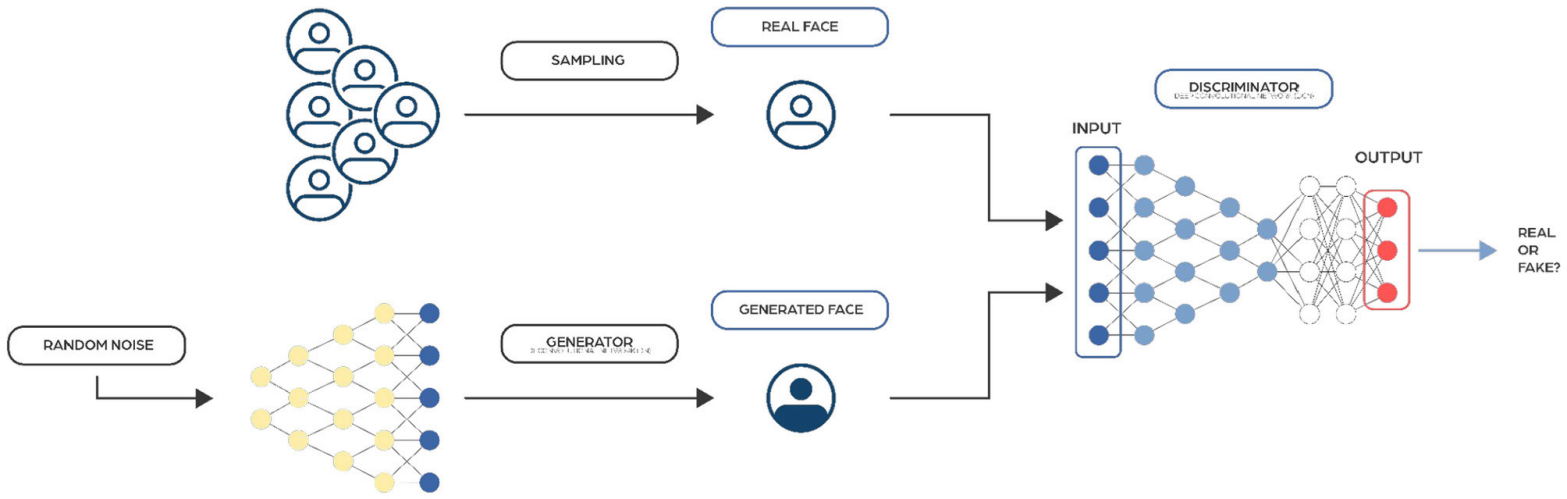
Image representing a typical Generative Adversarial Networks architecture. They consist of two components: the Generator, which creates synthetic data, and the Discriminator, which evaluates the authenticity of the data.

Generative Adversarial Networks (GANs) (Goodfellow et al., 2014) are made up of two interconnected networks, typically a combination of feedforward and convolutional neural networks (Figure 7). The basic idea behind GANs is based on the min-max two-player zero-sum game, where one player's gain is equivalent to the other player's loss. In GANs, these players are two networks called the generator (which input is most likely a random noise vector) and the discriminator (which input is real data samples from the training set and fake data samples generated by the generator). The primary goal of the discriminator is to identify whether a sample is derived from a fake or real distribution. Conversely, the generator's objective is to trick the discriminator by creating fake samples.

In microbiome research, GANs are helpful for tasks like increasing data variety, filling in missing data, and correcting inconsistencies across data batches. They create synthetic microbiome data used to test and improve statistical and machine learning models, making these models more diverse and reliable. Some advanced GANs, known as Conditional GANs (CGANs), can also include additional information like disease conditions to make the synthetic data more relevant and useful. Furthermore, GANs are effective at reducing batch effects (variations that occur when different groups of data are collected) while maintaining important features that are specific to particular diseases, improving the accuracy of disease detection and model performance.

#### Augmentation

Different publications used GAN architectures to design synthetic microbiome datasets that could be further used by other DL or ML algorithms. Rong et al. (2021) designed an algorithm based on GAN to simulate microbiome data that could be used to test statistical methods. Reiman and Dai (2020) implemented a modification of traditional GANs, the conditional GAN (CGAN) and added side information like disease or healthy state to the subject, generating samples with different distributions and increasing diversity. Oh and Zhang (2022) developed a Wasserstein GAN (WGAN) augmentation system, based on image data (the networks include convolutional layers to handle them). They clustered data before the augmentation of the metagenomic profiles in a visual pattern that is then augmented by multiple GANs. The visualization of the genomic data helps the network to catch more information, which is enough to enhance the performance of the prediction models, even on data not previously used for training, improving reproducibility.

#### Imputation

Choi et al. (2023) designed a Bidirectional RNN-based (BiRNN) GAN model to input missing data on a longitudinal study. Their model, DeepMicroGen, first uses CNN to extract features from microbiome data, imputing it to the BiRNN that acts as a generator. Afterwards, a Long Short Term Memory Networks (LSTM) RNN is used as a discriminator of the GAN model, identifying if a sample is authentic or imputed and its position in the study's timeline. Its evaluation in a real-case study with missing values demonstrated that the model could help fill in the gaps of this kind of study.

#### Batch correction

As previously described, Li et al. (2023) designed a DL-based algorithm based on GAN networks for this purpose (see Batch effect section). Their algorithm reduced the batch effect and improved the disease discrimination in 34 published studies. In addition, its combination with other classification algorithms, like RF, also improved the outcome of these models.

### Deep residual networks

Deep Residual Networks (DRNs) (He et al., 2015) are complex feedforward neural networks (FFNNs) that incorporate additional connections to transfer input from one layer to a subsequent layer, typically 2 to 5 layers ahead. These networks enforce an identity mapping by learning the relationship between an input and its corresponding output along with the original input. DRNs have exhibited effectiveness in recognizing patterns in architectures up to 150 layers deep (ResNet150). DRNs could potentially have the same applications as CNNs.

#### Microbiome interactions

DRNs have been employed in microbiome analysis to predict microbiome community composition. For instance, Michel-Mata et al. (2022) develop cNODE algorithm. This algorithm is able to predict taxa abundances in a community from the relative abundances of few training samples, instead of requiring complex time series of absolute abundance data to develop population dynamics as previous models. Although it presents some flaws, as not being able to predict abundance of taxa never seen or the lack of mechanistic interpretation, it could be a great instrument to infer how changes in microbial populations, like introducing species in a community with a fecal transplantation or changes due to antibiotic treatments, may affect the community composition.

### Attention networks and transformer

**Figure 8:**
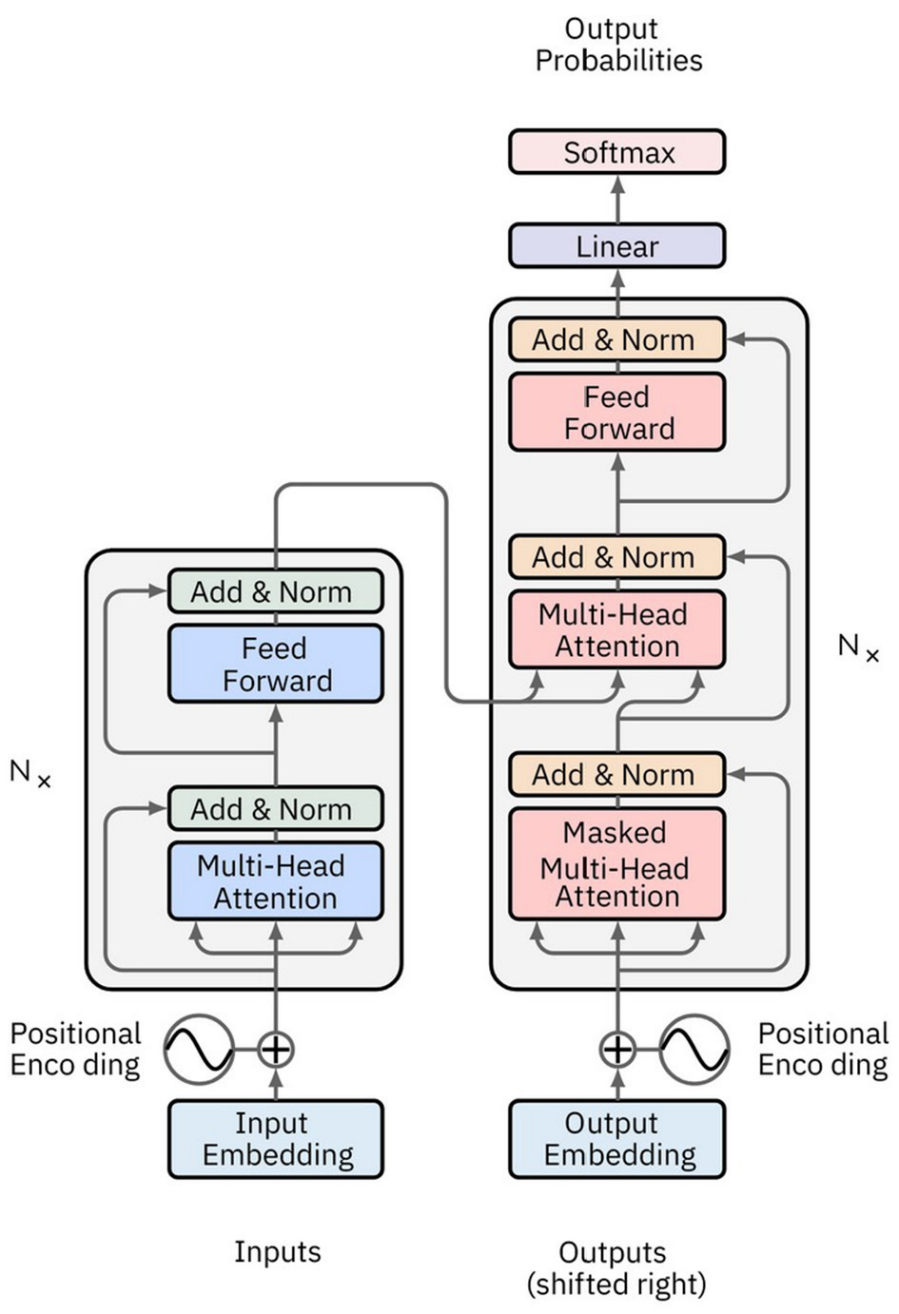
Image depicting a typical Transformer network architecture. The encoder consists of multiple layers, each with a multi-head self-attention mechanism and a feedforward neural network. The decoder also has multiple layers, each incorporating an additional attention mechanism that attends to the encoder's output. Positional encoding is added to the input embeddings to retain the order of the sequence.

Attention Networks (AN) address information decay by storing prior network states and allocating attention between these states. Encoding layers preserve hidden states in memory cells for each iteration. Decoding layers are linked to the encoding layers and also receive context-filtered data from memory cells. This filter enriches the decoding layers with the contextual importance of certain features. The attention network that generates this context is trained via the error signal from the decoding layer's output. Visualizing the attention context provides insights into the relationship between input and output features. Transformer networks, a type of AN introduced by Vaswani et al. (2023), rely exclusively on self-attention mechanisms instead of traditional RNNs (Figure 8). This approach enables them to effectively handle long-range dependencies. The architecture features an encoder-decoder structure, where both encoder and decoder consist of multiple layers of self-attention and feedforward neural networks. Each encoder layer comprises a multi-head self-attention mechanism followed by a position-wise feedforward network, with residual connections and layer normalization applied at each sub-layer. Decoder layers include an additional attention mechanism that attends to the encoder's output, facilitating tasks like sequence-to-sequence translation.

#### Functional annotation and metagenome-assembled genomes

Transformer models have been adapted for gene prediction and functional annotation in metagenomic datasets. Their self-attention mechanism captures complex dependencies between nucleotide sequences, enhancing gene identification accuracy compared to traditional methods. For example, MetaTransformer (Wichmann et al., 2023) employs these architectures to improve metagenomic sequence annotation, facilitating the discovery of novel genes and pathways.

#### Feature extraction and engineering

Transformers overcome other models in generating rich, context-aware embeddings representing microbial abundances, functional profiles, and environmental metadata. These embeddings are valuable for downstream tasks like clustering, visualization, and integration with other omics data. In this regard, MetaTransformer (Wichmann et al., 2023) uses Transformer-based embeddings to integrate metagenomic and metabolomic data, enhancing the interpretability and predictive power of microbiome studies.

#### Classification/prediction

Transformers excel in classification tasks by effectively modeling relationships within high-dimensional microbiome data. They are used to classify microbial communities based on taxonomic profiles, predict disease associations, and forecast environmental impacts on microbiomes. For instance, previously mentioned model MetaTransformer (Wichmann et al., 2023) leverages Transformers to predict microbial community shifts in response to environmental stressors, achieving higher accuracy than conventional machine learning models.

#### Microbiome interactions

The best example of AN in this application is found in Melnyk et al. (2023) where they tried to understand microbial community interactions using a combination of Attention mechanisms and other architectures (see Multi-architecture designs section). Moreover, Transformer-based models have been used to analyze interactions between microbial species and their metabolites, identifying key interactions that drive community structure and function. Thus, providing insights into microbial ecosystem stability and resilience. Whole Genome Transformer by Li Z. et al. (2024) exemplifies this approach by modeling gene interaction effects in microbiome habitat.

### Bidirectional encoder representations from transformers

**Figure 9:**
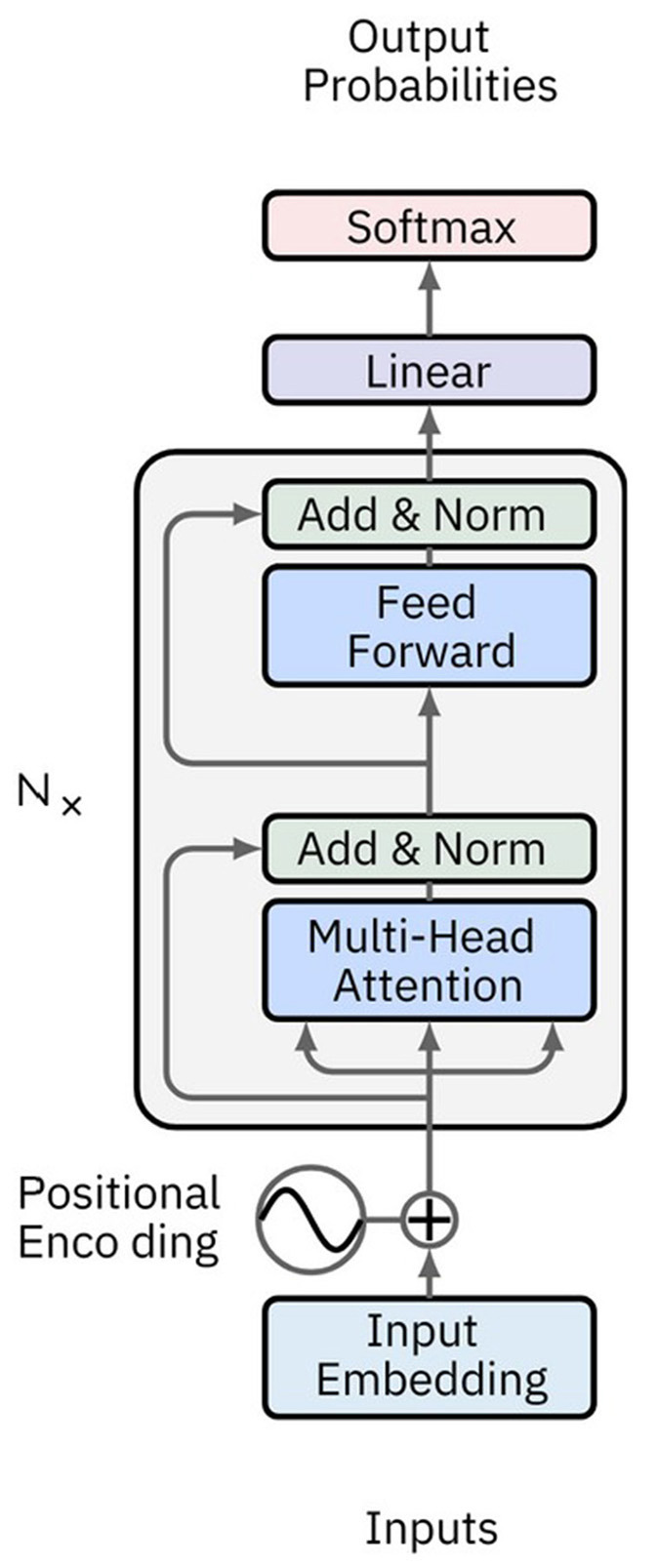
Image showing a classical BERT network architecture. BERT consists of multiple Transformer encoder layers with bidirectional self-attention mechanisms. Input tokens are embedded with positional encodings and passed through the encoder layers to produce contextualized embeddings.

BERT, introduced by Devlin et al. (2019) is a Transformer-based model designed for natural language understanding tasks. It employs bidirectional training of Transformer encoders, enabling the model to consider both left and right context in all layers. BERT is pre-trained on large text corpora using two unsupervised tasks: Masked Language Modeling (MLM), which predicts masked words within a sentence, and Next Sentence Prediction (NSP), which assesses relationships between sentence pairs. This pre-training allows BERT to generate rich contextual embeddings that can be fine-tuned for various downstream tasks with relatively small labeled datasets (Figure 9).

#### Functional annotation and metagenome-assembled genomes

BERT-based models have been adapted for functional annotation by treating gene sequences similarly to natural language. Leveraging bidirectional context, these models can more accurately predict gene functions and interactions. For instance, DNABERT (Ji et al., 2021) applies BERT to nucleotide sequences to improve the identification and annotation of functional genes within metagenomic assemblies, outperforming traditional annotation tools in accuracy and speed. In addition to this, hierarchical or small BERT models (Zhang Y. et al., 2022; Abdelkareem et al., 2018; Gwak and Rho, 2022) have been utilized not only for this purpose but also for identifying antimicrobial peptides (Ma et al., 2022), as well as predicting gene or protein domains (Zhang Y. et al., 2022).

#### Feature extraction and engineering

BERT excels generating contextual embeddings. For example, DNABERT (Ji et al., 2021) uses BERT-derived embeddings to integrate metagenomic, metabolomic, and environmental data, facilitating comprehensive feature engineering for microbiome studies.

#### Classification/prediction

BERT's ability to generate context-aware embeddings makes it highly suitable for classification tasks in microbiome research. It has been used to classify microbiome samples based on disease states, environmental conditions, or treatment responses. BioBERT (Lee et al., 2020) has been fine-tuned for microbiome samples, achieving superior performance in classifying conditions such as inflammatory bowel disease (IBD) and obesity compared to standard machine learning classifiers.

#### Microbiome interactions

BERT-based models use bidirectional attention to model interactions between different microbial species and their metabolites. This approach identifies key interaction networks that influence community structure and function, providing deeper insights into microbiome ecology. Whole Genome Transformer (Li Z. et al., 2024) exemplifies this by modeling gene interactions within microbial habitats.

### Kohonen networks or self organizing maps

Kohonen Networks (KN), also known as Self Organising Maps (SOM) (Kohonen, 2013), leverage competitive learning for unsupervised data classification. The network determines which neurons closely correlate with the input upon receiving input. These neurons are subsequently adjusted to better match the input, influencing neighboring neurons. The degree to which neighboring neurons are adjusted depends on their proximity to the best matching units, integrating the spatial information in the learning process. In microbiome analysis, SOM methods enable clustering and visualization of genes from individual species with much higher resolution than traditional methods like principal component analysis, providing insights into the molecular mechanisms underlying genome signatures (Iwasaki et al., 2013).

### Graph neural networks

**Figure 10:**
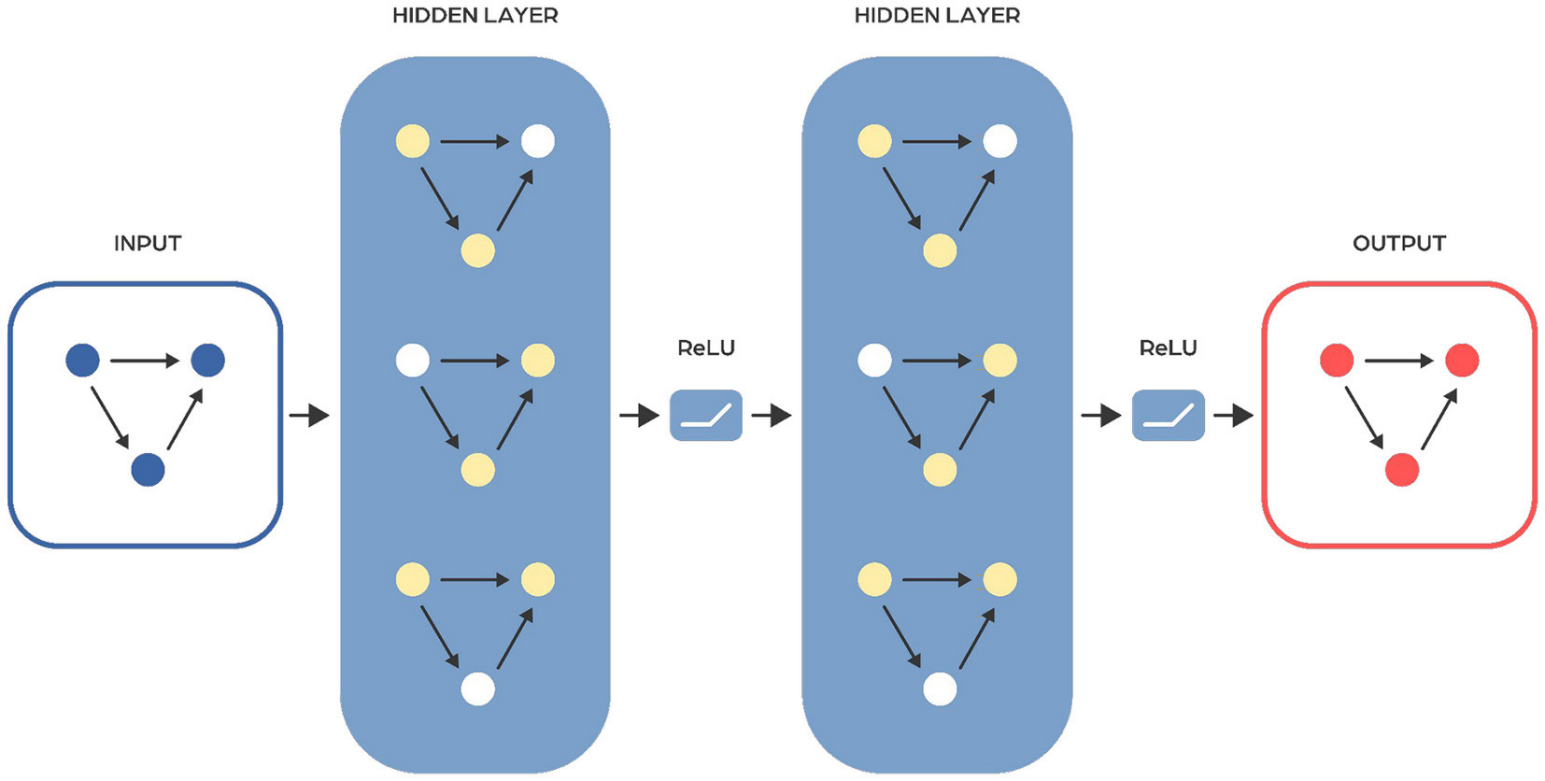
Graph Neural Networks architecture. Each node in the graph represents an entity, and edges represent relationships between entities. The architecture includes layers that aggregate and transform information from neighboring nodes, enabling the network to learn representations that capture the graph's structural and feature information.

Graph Neural Networks (GNNs) (Scarselli et al., 2009) are designed to process data structured as graphs, effectively capturing complex relationships among data points (Figure 10). Unlike conventional networks that require data inputs to be organized in a grid-like manner (such as images or sequences), GNNs exploit the intrinsic properties of graphs, making them suitable for irregular and complex data structures. By using node and edge representations, GNNs propagate information across nodes, allowing each node to aggregate and process information from its neighboring nodes (Lamurias et al., 2022). Over iterations, nodes gradually develop high-level representations embodying local and global structural information, enhancing their capability to perform tasks like node classification, link prediction, or graph classification. The input to a GNN typically consists of graph structure (e.g., adjacency matrix), node (and sometimes edge) features.

#### Functional annotation and metagenome-assembled genomes

Lamurias et al. (2022) implemented GraphMB, a NN that leverages the GNNs properties to use them in the metagenomic binning process. It uses the graph originated from the assembly process to generate embeddings retaining node-neighbors information.

#### Features extraction and engineering

Zhu et al. (2019) explored the possibility of using GNNs for feature selection. Using relative abundances, they generated correlation networks that were fed into feedforward networks. This strategy allowed them to identify the key taxa that are driving that microbial community.

#### Classification/prediction

Some groups have tried to develop classification algorithms taking advantage of GNNs power. That is the case of Jiang et al. (2023) or Khan and Kelly (2019) who developed a Graphical network for multiclass disease prediction, being able to distinguish between 19 different diseases better than a classical FFNN.

#### Multi-view analysis

MOSDNET is a multi-omics classification framework that effectively extracts shared and specific representations from different omics data (Li et al., 2024). This framework leverages Simplified Multi-view Deep Discriminant Representation Learning (S-MDDR) and Dynamic Edge Graphical Convolutional Network (DEGCN) to enhance the accuracy and efficiency of disease classification.

### Natural language processing models

Human languages are a set of symbols combined following certain rules that allow us to encode information. Natural language processing or NLP is an area of AI and DL that includes all techniques and mechanisms to favor the understanding of natural (not mathematics or other variants) human language, the decoding of the information they contain, by computers. This has been an exploding sector in recent years thanks in part to the development of DL and its powerful inference capacity. Also, to the applications in NLP of different models like Transformer (Vaswani et al., 2023), based on attention mechanisms, that looks for connections in the different elements of the data (words in this case) and coherence inside those connections. Microbiome sequences encode their information into the combination of 4 symbols, 4 nucleotides that favor that information storage. Thus, NLP appeared here as a clear instrument to make sense of and interpret those sequences, extracting the information that allows the microbiome to generate enzymes, establish relationships and survive in its environment.

#### Functional annotation and metagenome-assembled genomes

The NLP models have been adapted to annotate metagenomes recently. Word embedding techniques, which involve embedding k-mers alone (Mock et al., 2022; Arango-Argoty et al., 2021) or in combination with other deep learning architectures like CNN or LSTM (Shang and Sun, 2021; Miao et al., 2023; Liu et al., 2023), have also been used to detect viral genomes. Finally, DeepMicrobes (Liang et al., 2020) using LSTM architecture and self-attention models or models using the word2vec method to combine k-mer embeddings with taxonomy, like NLP-Me taxa (Matougui et al., 2021), FastDNA (Menegaux and Vert, 2019), or Metagenome2Vec (Queyrel et al., 2020) are some approaches to metagenomic profiling and taxa identification. However, they have not managed to beat other models described earlier (based on VAE), mostly due to the difference between small k-mers (3, 4 bases) and words, and the high computational demands that increasing k-mers longitude involves. These issues lead the way toward other models tackling those weaknesses. In this sense, META^2^ (Georgiou et al., 2019) and BRUME (Menegaux, 2020) have tried grouping in the same encoding (thus reducing computational requirements) longer k-mers by proximity, while others are applying different NLP methods like LDA (Latent Dirichlet Analysis) or LSA (Latent Semantic Analysis) (Tran et al., 2022).

#### Features extraction and engineering

Some NLP models with word embedding, like GloVe (Roy et al., 2024), have been also applied in the microbiome feature extraction process. Tataru et al. (2022) generated an R package (GMEmbeddings) where using this model and studies from the American gut project, calculated a “translation” (embedded) matrix that can be applied to any other 16S study to generate a new embedded matrix in that study for further analysis. Thus, they aimed to reduce the batch effect occurring in predictions when using samples from different studies at the same time, and generate a tool that takes and reduces information from several studies and can then be applied to favor the reproducibility (by “homogenizing” embeddings) in the analysis and prediction of other datasets. Additionally, these methods are employed for embedding, interpreting categorical variables, and representing them as continuous vectors like in Shang et al. (2022) and Ma et al. (2022), where natural language processing is used over microbiome data to identify bacteriophages and antimicrobial peptides respectively.

### Multi architecture designs

Several groups have aimed to exploit the advantages and strengths of different architectures, while diminishing their weaknesses, by combining different models into the data analysis to achieve better results in their microbiome projects. In this section you can find multi architecture designs applied to some of the tasks described previously. Although the combined models may involve high computational demand, the results sometimes surpass the mere use of simpler models especially under complex experimental designs or heavy preprocessing of the datasets.

#### Functional annotation and metagenome-assembled genomes

Zhao et al. developed Read2Pheno (Zhao et al., 2021) a multi-architecture network tailored to provide features information and disease prediction directly from reads. To do so, they combined CNN, RNN and attention mechanisms. Thus, they were able to leverage the information of thousands of reads for few samples, predicting from the reads directly: (I) taxonomy levels present in the sample, (II) microbiome phenotype (origin in the body for that microbiome), and (III) host phenotype (disease diagnosis).

#### Microbiome interactions

Melnyk et al. (2023) combined graph algorithms, applied to microbial communities, with Transformer (Vaswani et al., 2023), a neural network based exclusively on attention mechanisms, to obtain lower-dimensional representations of the bacterial communities. They also used Layer-wise Relevance Propagation (LRP) (Bach et al., 2015) to interpret the decisions made in a NN. This helped to understand the evolution of changes over time by retaining the metastable properties of those communities and to find patterns in the generated graph neural networks that could highlight dynamics in the community pointing toward a change in the metastability from a healthy to a disease state.

#### Classification/prediction

The combination of different NN architectures has proved as a valuable strategy to improve classification tasks regarding metagenomic analysis. Ditzler et al. (2015) evaluated various neural network architectures, including MLPNN, RNN, and a Boltzmann machine, on two different datasets for classifying bacteria regarding pH and body location comparing them with a RF classifier. Their findings indicated that the MLPNN was competitive enough against the RF, while the other deep methods could not match RF performance. However, a possible advantage over larger datasets for these deep learning approaches was suggested. Lo and Marculescu (2019) designed two models using FFNN and CNN with previous data augmentation by generating new samples with a known distribution (negative binomial distribution) and adding Dropout layers to the architecture to prevent overfitting. This approach was designed to deal with the problem of limited availability of large datasets.

Mulenga et al. (2021) proposed a combination of feature extension (combining different normalization methods) and data augmentation (using VAEs) previous to a FFNN architecture to improve the classification outcome. Oh and Zhang (2020) used autoencoders to distill data to a lower-dimensional state to reduce potential noise in the data and retain the important features. This processed data was then used as input for various machine learning models, including SVM, RF, and deep learning models like MLPNN. Although MLPNN did not always beat the other DL methods, the use of autoencoders was revealed as a great addition for improving healthy disease/classification. Moreover, the same group of researchers made another attempt to increase NN performance with augmentation by linking the classification layer to a prior GAN system. This GAN augmentation system based on the recognition of visual patterns in metagenomic data (Oh and Zhang, 2022), significantly improved the classification of previously unseen data. The synergy of this system with both NN and SVM algorithms demonstrated superior performance compared to similar classifiers.

Zeng et al. (2022) used shotgun metagenomic data to provide taxonomic and functional information to a neural network combining CNN and LSTM-RNN models. After reshaping the functional information to 2D arrays and reducing the dimensionality of taxonomic information by clustering, they performed a joint prediction directed to unveil the “theater of activity of the microbiome.” They also provided feature analysis by providing both raw information sources to a LSTM network combined with SHAP (Lundberg and Lee, 2017) algorithm for explainability. In a similar way Sharma and Xu (2021) used CNN to extract features from input data combined with LSTM, which retains the important information that the input had. Notably, they introduced a time series component where new measurements from the same patients were added to the CNN, integrating this new input with the previous LSTM output. This combination of NN achieved an efficient system by merging the feature extraction of the CNN with the ability to retain sensitive information through timepoints of the LSTM. Fung et al. (2023) also combined CNN and LSTM architectures together with self-knowledge distillation, where the network learns from itself by taking into shallow sections information from the deeper parts, to perform disease prediction. This design proved to overcome other networks trained on longitudinal studies.

#### Multi-view analysis

Liu M. et al. (2022) used a DL approach by designing a complex algorithm, e-DeepBGC leveraging NLP in the identification of BGCs. This model includes different protein family domain (Pfam) information embeddings, CNN, BiLSTM, and data augmentation between training epochs by synonym replacement of Pfams and random shuffling to generate artificial genomes. This architecture outperformed the prediction accuracy of all state-of-the-art models, including the previous version of itself, the DeepBGC (Hannigan et al., 2019). In addition, DeepIDA-GRU is a pipeline that utilizes both statistical and deep learning techniques to integrate cross-sectional and longitudinal data from various sources (Jain and Safo, 2024). This pipeline includes several key components: variable selection and ranking using both linear and nonlinear methods, feature extraction through functional principal component analysis and Euler characteristics, and joint integration and classification. For cross-sectional data, it employs dense feed-forward networks, while recurrent neural networks are used for longitudinal data.

## Other architectures and novel trends

The below listed architectures have limited (or no) examples of application in the microbial sciences.

### Liquid state machines and echo state machines

Liquid State Machines (LSMs) (Maass et al., 2002) and Echo State Networks (ESNs) (Jaeger, 2001) are specialized types of recurrent neural networks. LSMs, as spiking neural networks, use threshold functions instead of sigmoid activations, with each neuron acting as an accumulating memory cell that triggers a spike when a threshold is reached. ESNs, in contrast, have random inter-neuronal connections and employ a unique training method where only the output layer connections are adjusted over time, while input data primes the network.

It is unlikely that those models will be widely used in microbiome analysis due to their specific architecture and limitations for handling microbiome data. These models rely on recurrent neural networks with fixed random connections, which may not effectively capture the complex dynamics and relationships present in microbiome datasets. Additionally, these architectures often require careful tuning of parameters and may not offer significant advantages over more conventional machine learning approaches for microbiome research.

### Neural turing machines and differentiable neural computers

Neural Turing Machines (NTMs) (Graves et al., 2014) are an advanced form of LSTMs that separate memory from neurons, combining neural network expressiveness with digital storage efficiency. NTMs use a neural network to interact with a content-addressable memory, making them Turing complete by enabling read, write, and state alteration functions.

Differentiable Neural Computers (DNCs) (Graves et al., 2016) improve upon NTMs by using RNNs to manage scalable memory, inspired by the human hippocampus. DNCs incorporate attention mechanisms to query input similarity, temporal memory relationships, and update recency for memory management.

However, due to their complexity, high computational demands, and specialized nature, NTMs and DNCs are unlikely to be widely adopted in microbiome analysis.

### Capsule networks

Capsule Networks (CapsNet) (Sabour et al., 2017) represent an alternative to pooling in neural networks, inspired by biological systems. Unlike traditional neural connections that utilize a single weight (scalar), CapsNet employs multiple weights (vector), enabling the transfer of comprehensive information, including the detected feature's attributes like location, color, and orientation within an image. The network's learning algorithm integrates a localized form of Hebbian learning that emphasizes the importance of accurate output predictions in subsequent layers. We are not aware of any biological applications of capsule networks, but their unique features (like the ability to model complex, hierarchical feature representations, and in particular preserving spatial relationships) could enable us to disentangle and tackle the complexities of human disease.

### Kolmogorov-Arnold networks

Kolmogorov-Arnold Networks (Liu et al., 2024) are neural networks based on the Kolmogorov-Arnold superposition theorem. This theorem states that any continuous function of multiple variables can be represented as a combination of functions of one variable. In neural networks, this concept involves decomposing complex functions into simpler components, which are then combined to approximate the original function. Kolmogorov-Arnold networks use this approach to learn and represent complex mappings between input and output data, making them effective for handling diverse and high-dimensional data in deep learning applications.

Although this approach is relatively new, Kolmogorov-Arnold Networks are expected to become more popular in microbiome analysis because they effectively handle complex data with many variables. They achieve this by breaking down these complicated functions into sums and combinations of simpler, single-variable functions, a method based on the Kolmogorov-Arnold superposition theorem. By simplifying complex functions into one-variable components, these networks speed up computations and make the results easier to interpret.

### CRISPR guide RNA

CRISPR technology offers promising tools for microbiome engineering through targeted genetic modifications (Ramachandran and Bikard, 2019; Bai et al., 2023), while deep learning methods assist in refining target selection and optimization for potential therapeutic use. Deep learning has significantly enhanced the design of guide RNAs (gRNAs) for CRISPR/Cas12a-based diagnostics by enabling precise prediction and optimization of gRNA efficiency and specificity (Lee, 2023). Traditional gRNA design often struggles with off-target effects and variability in cleavage efficiency, which can compromise diagnostic accuracy. Deep learning models, trained on large datasets of sequence-function relationships, can predict gRNA binding affinity, cleavage activity, and off-target risks with high precision. These models account for sequence context, secondary structure, and thermodynamic properties, enabling the design of highly effective gRNAs tailored to specific targets. In the context of diagnostics, optimized gRNAs improve the sensitivity and specificity of CRISPR/Cas12a systems for detecting nucleic acids, critical for applications such as pathogen detection, genetic disorder screening, and environmental monitoring (Huang et al., 2024; Chuai et al., 2018; Zhang et al., 2023). By leveraging deep learning, researchers can accelerate the development of robust and scalable diagnostic tools, addressing diverse biological and medical challenges.

#### Notable advancements include

DeepCRISPR developed by Ramachandran and Bikard (2019), a comprehensive computational platform to unify sgRNA on-target and off-target site prediction into one framework with CNNs. Liu et al. (2024) developed a deep learning model based on CNNs called EasyDesign to facilitate rapid and highly efficient crRNA design for Cas12a-based detection. Zhang et al. (2023) developed three deep learning models (AIdit_ON, AIdit_OFF, and AIdit_DSB) for predicting the cleavage activities, editing specificities, and repair outcomes of SpCas9/gRNA.

## Risks and considerations

In the dynamic field of microbiome research, deep learning faces several critical challenges, each impacting the reliability and applicability of research outcomes. Here we describe in depth the most important challenges associated with DL in microbiome. You can also find a shorter summary table with examples on microbiome data of the most common risks and potential solutions that can be implemented (Supplementary Table 4).

### Model overfitting

Overfitting (Lever et al., 2016) is a significant challenge in deep learning, particularly prevalent in microbiome research where small datasets are common. This issue, where a model learns too much from the specifics and noise of its training data, compromises its ability to perform effectively on new, unseen data. Various deep learning applications in microbiome research, including GANs like DeepMicroGen (Choi et al., 2023), and GAN-GMHI (Li et al., 2023), are particularly susceptible to overfitting. When trained on limited datasets, these models tend to capture noise, resulting in less effective generalization. Moreover, this can lead to misleading conclusions about the relationships within microbiome data.

Regularization and careful network architecture design are crucial to address overfitting. Autoencoders, used for predicting shifts in microbiome communities (Reiman and Dai, 2019), also face overfitting risks, which can be mitigated through dropout techniques and sparse autoencoder implementation. Similarly, in the analysis of large-scale microbiome data, Batch-Learning Self-Organizing Maps (BLSOMs) can help mitigate overfitting by aligning map size with dataset complexity and incorporating regularization (Iwasaki et al., 2013).

### Interpretability

Interpretability (Teng et al., 2022) of models in machine learning, particularly in healthcare, refers to the ability to understand and explain how and why a model makes its predictions. It involves deciphering the model's decision-making process, making it transparent and understandable to humans. This is crucial because it builds trust in the model's predictions, ensures compliance with state of knowledge and regulatory standards, and aids in the clinical decision-making process. DL is known for its “black box” nature, which can obscure insights and imposes challenges with interpretability and reproducibility. e.g., 16S rRNA sequencing data from fecal samples of T2D (Pfeil et al., 2023) patients and healthy control subjects served to identify relative abundances of thousands of bacterial taxa. Preprocessing, including removal of low-quality reads and contaminants, normalization and feature selection, enabled focusing on specific and relevant bacterial taxa known or hypothesized to be associated with diabetes. To avoid reproducibility issues at this stage, it is important to track every change in the data to find out the impact of different preprocessing methods like dimensionality reduction, etc. Regarding interpretability this is much more difficult, since many methods, such as PCA, have low interpretability. This lack of transparency can be problematic when researchers need to understand which specific features (e.g., particular microbial taxa or genes) are driving the associations with disease states or treatment outcomes.

For our example, the most commonly chosen DL model is CNN, which usually includes several convolutional and pooling layers to extract and learn the most relevant features from the microbiome data, followed by one or more densely connected layers for classification. Therefore, any changes made to the model, such as the number of layers or hyperparameter values, must be trackable. The standard solution for tracking the evolution of the code is to use a version control system such as Git, which can provide the required reproducibility. The same applies to training and validating the model to monitor its performance and avoid overfitting. However, it creates problems related to lack of interpretability. The CNN model acts as a black box, making it impossible to understand how specific features (taxa) affect the prediction, which is challenging due to the many levels of transformation and non-linearities. The convolutional and dense layers might capture complex interactions between different bacterial taxa, but these interactions are not readily interpretable or easily mapped back to biological insights. In addition, there are what are known as generalization concerns: Without clear insights into what the model is “learning,” there is a risk that the model will not generalize well to other datasets or populations and may capture artifacts or biases in the training data. Therefore, its utility in providing interpretable insights for scientific understanding or even clinical decisions is limited.

One of the possible solutions to deal with interpretability issues is integrating explainable AI (XAI) techniques. These techniques help to uncover the reasoning behind model predictions, making the models more transparent and their findings more actionable in a scientific and clinical context. For example methods like Layer-wise Relevance Propagation (LRP) (Bach et al., 2015), SHAP (SHapley Additive exPlanations) (Lundberg and Lee, 2017), or LIME (Local Interpretable Model-agnostic Explanations) (Ribeiro et al., 2016) can provide explanations for individual predictions based on approximations, showing how each feature contributes to the output for a specific sample. This was intended to provide a better understanding of how different microbial compositions influence disease prognosis. For example, LRP works by propagating the prediction backward through the network layers, assigning a relevance score to each neuron and ultimately to each input feature. This process highlights which features have the most significant impact on the model's output. SHAP is a flexible framework based on cooperative game theory that offers consistent and locally accurate explanations of feature importance for any deep learning models. It works by calculating Shapley values, which represent the average contribution of each feature across all possible combinations of features. LIME is a technique that helps interpret the predictions of complex deep learning models by approximating them with simpler, human-readable models.

Nevertheless, it appears that these models do not always show reliable results in complex models. First, the approximations might not always capture the true underlying relationships, especially in highly non-linear or interaction-heavy models. Moreover, there is a risk of overinterpreting the outputs of models, especially if the nuances of how these methods generate explanations are not fully understood. In addition, explanations can sometimes be unstable, with small changes in the input data leading to significantly different explanations. One possible strategy to address these issues could be to combine different interpretability techniques and sanity checks that provide a more comprehensive understanding of model behavior.

Another approach to overcome the black box is to develop more intuitive visualization tools that can help interpret model outputs and make these methods more accessible to non-experts. Pfeil et al. (2023) used a radial heatmap to visualize classified microbiome sequencing data, which resulted in a discrimination accuracy of 96%. Different visualizations at the genus level were used for training and classification to check robustness and generalization potential. The applied cross-validation and the comparison between validation and test set revealed no particularly advantageous visualization. This method contributes significantly to interpretability and could potentially be used to predict other diseases. Finally, the multimodal variational information bottleneck (MVIB) from Grazioli et al. (2022) proposes as an approach in microbiome the integration of multiple heterogeneous data modalities into a unified disease prediction framework. This integration provides a more comprehensive understanding of the microbiome's role in various disease states. Its ability to classify diseases effectively, as demonstrated through its application to diverse disease cohorts, is complemented by its interpretability. MVIB employs a saliency technique, allowing it to identify the most relevant microbial species and strain-level markers in making predictions. This interpretability is invaluable, offering insights into the specific microbial factors associated with diseases and guiding more targeted therapeutic strategies (Lundberg and Lee, 2017).

### Data leakage and information leakage

Data leakage in machine learning happens when a model accidentally gets access to information that it shouldn't have or sensitive information could be extracted from the model. The extent of the potential damage depends on the type of leakage, where we can distinguish two main types.

1. **Data Leakage** where a model accidentally gets access to information from the validation or test sets (Chollet, 2021). This can occur if, for example, the entire dataset is preprocessed before splitting it into the different data sets. Thus, causing information from the test set to influence the training data. Another common source is tuning model hyperparameters based on test set performance, which means the model is indirectly learning from data. In such a case the test set no longer serves as an independent evaluation of the model's performance, resulting in biased and misleading performance metrics (Chen et al., 2020). This leakage is a significant problem because it leads to overly optimistic performance metrics; the model appears to perform better than it genuinely does because it has effectively “seen” the answers in advance. As a result, the model may not generalize well to new, unseen data, defeating the purpose of building a predictive model. It might perform exceptionally on the validation or test sets but fail in real-world applications where it encounters truly new data. To deal with this problem, it is crucial to carefully separate your dataset into three distinct sets: training, validation, and test sets. The training set is used to fit the model, the validation set is used to tune hyperparameters and make decisions about the model architecture, and the test set is reserved strictly for the final evaluation after all tuning is complete. By ensuring that the model doesn't have access to the validation or test data during training, you prevent information from leaking and obtain a more accurate assessment of the model's true performance.

2. **Information Leakage** where sensitive information can be extracted from models containing original data subjects/owners. This could happen, particularly through gradient inversion in deep learning (Hatamizadeh et al., 2023) and represents a significant risk in fields such as microbiome research. It usually can happen when samples are not properly randomized or when certain variables that correlate with the outcome are included in both datasets. This issue, involving the unintended exposure of sensitive medical data, is a major concern in the analysis of complex datasets. Susceptible to this risk are advanced deep learning models like Generative Adversarial Networks (GANs), autoencoders, and Transformers. These models, while effective in processing intricate data, can inadvertently reveal sensitive information, especially if the learning gradients are exposed. The implications of data leakage in medical applications are substantial. For instance, in studies employing models like TaxoNN for disease prediction (Sharma et al., 2020) or deep representation learning techniques (Melnyk et al., 2023), the unintended exposure of patient-specific microbiome data could result in privacy violations, breaching confidentiality and raising legal and ethical issues. The highly personalized nature of microbiome data amplifies the need for stringent measures to prevent such leakage. To mitigate this risk, several strategies are being implemented. Differential privacy in deep learning models ensures that outputs do not disclose sensitive individual information, crucial in models that might learn identifiable patterns. Secure Multi-Party Computation facilitates collaborative deep learning without exposing individual data points, relevant in collaborative projects like multi-layer and recursive neural networks for metagenomic classification. Homomorphic encryption^1^ (Munjal and Bhatia, 2023) allows for computations on encrypted data, without having to decrypt it. The resulting computations are left in decrypted form, protecting sensitive information in deep learning applications, a vital approach in studies identifying antimicrobial peptides or bacteriophages. Moreover, establishing robust data sharing and processing protocols, including data anonymization and secure handling practices, is essential in large-scale studies for disease prediction or microbe-disease associations.

### Data imbalance

Data imbalance (Fang, 2023), where certain classes or conditions are underrepresented, can bias predictive models. This is evident in disease prediction studies like (Sharma et al., 2020), where models may favor the majority class. Moreover, generative models like MB-GAN (Rong et al., 2021), used for microbiome simulation or data imputation, also struggle with data imbalance. They may produce less diverse or skewed synthetic data, adversely affecting analyses and interpretations, especially in disease prediction and diagnosis. Additionally, data imbalance poses a challenge in capturing rare but significant microbiome events or features, potentially overlooking critical biological insights.

To tackle these challenges, the approaches demonstrated by DeepMicro (Oh and Zhang, 2020) and phyLoSTM (Sharma and Xu, 2021) provide effective strategies. DeepMicro, with its deep representation learning framework, addresses the high-dimensionality and sparsity of microbiome data, a direct outcome of data imbalance. On the other hand, phyLoSTM's novel approach of combining CNNs and LSTMs offers an advanced method to analyze longitudinal microbiome sequencing data. This model effectively manages variable time points in subjects and balances the weights between imbalanced cases.

### Other data biases

A prevalent issue in deep learning for microbiome research is data bias. This occurs when training data doesn't accurately reflect real-world scenarios, leading to skewed results. Biases in microbiome data that impact deep learning models arise from various stages of experimental and analytical workflows (Nearing et al., 2021). For example, sample collection methods can introduce biases based on how, when, and where samples are collected, leading to inconsistent microbial representation. DNA extraction protocols further contribute to bias since different microbes have varying cell wall strengths, resulting in unequal extraction efficiencies. Amplification biases during PCR can skew the observed abundance of certain microbes, as some DNA sequences amplify more efficiently than others. Sequencing platforms also introduce biases due to differences in error rates and read lengths. Additionally, bioinformatic processing, such as sequence filtering and taxonomic classification, can further distort the true microbial composition. In addition, models like phyLoSTM (Sharma and Xu, 2021) or DL-TODA (Cres et al., 2023), used for disease prediction, may perform inaccurately for underrepresented groups if trained on data from a specific population.

Consequently, even the most sophisticated models cannot produce reliable outcomes if trained on biased or poor-quality data, as this can lead to overfitting, reduced generalizability, and misleading predictions. Improving the reliability of deep learning applications in microbiome research requires diverse, representative training data and the application of fairness-aware machine learning techniques. Regular model auditing and interdisciplinary collaboration are also essential for effectively mitigating these biases.

Model Drift is a critical challenge in deep learning applications, where the performance of machine learning (and deep learning, in particular) models degrades over time due to changes in the underlying data or environment. This phenomenon is particularly prevalent in microbiome research, as the characteristics of microbial communities are subject to change due to environmental shifts, dietary changes, and other factors. Models like DeepMicroGen (Choi et al., 2023), used for microbiome simulation or data imputation, and disease prediction models like GAN-GMHI (Li et al., 2023) or DeepMicro (Oh and Zhang, 2020), are susceptible to accuracy loss as microbial landscapes and human-microbiome interactions evolve.

### Sample size

An article by Rajput et al. (2023) suggests that when using machine learning in microbiome research, an appropriate sample size of data is essential to obtain reliable results. The paper proposes two criteria. First, the sample should be large enough for the effect of its analysis to be significant [average or grand, of at least 0.5 according to Cohen's scale,^2^ a measure of effect size (Cohen, 1988)]. Second, the accuracy of machine learning models on this sample should be at least 80%, and additional data above this sample size should not significantly increase accuracy. In short, the idea is to find a “golden point” in sample size where additional data does not significantly improve accuracy. Still, the sample is large enough for the results to be reliable.

### Model benchmarking

Benchmarking is crucial for evaluating the performance of any computational method prior to its release. This is equally true for microbiome DL analysis methods, which require well-designed benchmarks to accurately reflect the diverse conditions in microbiome studies. Depending on the aim, benchmarking can assess various evaluation metrics, such as model performance, runtime, or memory usage. This can be done both in an absolute setting (only for a new method) or a relative setting, when comparing the method with other approaches (Bokulich et al., 2020). Several aspects need to be considered while creating the benchmark with the most important: selection of a representative test set, parameter tuning and selection of appropriate metrics.

Microbiome data presents unique challenges for benchmarking deep learning models due to its compositional nature, correlation between taxa, high dimensionality, and sparsity. Test data typically should allow measurements of method accuracy which mean we need to have a “ground truth” of some type, e.g., samples with known composition. Several types of data can be used for that purpose: mock data, biological data or simulated data (Bokulich et al., 2020). Mock data consist of mixtures of microbial cells mixed at known ratios and their taxonomic identities (Dale et al., 2018; Highlander, 2013). As the known composition of mock data makes, they are frequently used in microbiome benchmarking. However, since they require running experiments, they are expensive to generate and often of limited availability. Biological data also come from experiments but they are typically not measured for the purpose of testing methods. Analysis of such data then requires accounting for all challenges related to preprocessing measurements coming from real operating conditions. There are many repositories of such datasets such as NCBI-SRA, European Nucleotide Archive or Qiita (Gonzalez et al., 2018) but the main issue with using such data is that we do not have objective truth to compare with. Finally, simulated data are cheap to generate compared to mock or biological data. However generating the realist dataset is challenging as methods need to take into account the characteristics of the microbiome data such as correlation between taxa, sparsity, overdispersion, and compositionality (He et al., 2024). Ideally, for benchmarking purposes, various different datasets should be analyzed, as different sample types (for example gut vs. soil) can be characterized by different microbial diversity. By using many different dataset one can avoid overfitting the method to a particular type of a sample.

Training DL models usually involves searching for model best hyperparameters and finding such parameters can significantly impact the model performance. In contrast to the classical machine learning approach in deep learning, hyperparameter tuning is often more critical due to the complexity and depth of neural networks. Deep learning models have many hyperparameters, such as learning rate, batch size, number of layers, and types of activation functions (Li et al., 2022). Proper tuning can significantly impact the models performance and convergence, and is essential for achieving high accuracy and generalization. However, when performing parameter tuning it is crucial to avoid introducing bias and apply tuning procedure to all the methods which are used in comparison with a new method (Weber et al., 2019). The researchers should not assume that when comparing with other methods, they should be applied with “out-of-the-box” parameter settings as applying any ML or DL model to a practical problem requires tuning its hyperparameters to fit a specific dataset. This can pose a significant challenge, as given the large number of parameters and the non-linear nature of deep learning models, finding the optimal set of hyperparameters can be extremely computationally expensive (Yang and Shami, 2020).

Selection of the proper metric is related to the task performed which typically is classification or regression. Microbiome datasets are typically multi-class datasets with highly imbalanced microbial communities and any applied metric should account for such data characteristics and be suitable to the specific problem. Usually more than one performance metric should be analyzed in order to avoid an implicit bias by so-called selective reporting by searchers testing their own method and thus providing over-optimistic results (Norel et al., 2011). As there were many works discussing the guidelines related to metrics for classification and regression tasks (Marcos-Zambrano et al., 2021; Hoffmann et al., 2019; Fischer et al., 2024; Liu et al., 2018) we will not cover this topic in depth here, but you can find a summary of the most commonly used evaluation metric in Supplementary Table 3. In addition to standard metrics used in machine learning settings, for the microbiome data, alpha-diversity (diversity within a community) or beta-diversity (diversity between communities) vs. the expected diversity can be used (Sinha et al., 2017; Wirbel et al., 2024). In a similar way, the values of alpha and beta diversity metrics can be compared with the expected diversity measurements for simulated or mock communities (Willis and Martin, 2022).

Considering the complexity of benchmarking deep learning models for microbiome studies, we have included a comparative table (Supplementary Table 5) summarizing the strengths and weaknesses of the models discussed in this review. This table is designed to assist end-users in making informed decisions when selecting models for their specific applications.

## Conclusions

Deep learning has the potential to revolutionize microbiome research by providing powerful tools to manage the complexity and high dimensionality of microbiome datasets. This review presents a comprehensive overview of deep learning applications in microbiome research, highlighting the capability of these methods to enhance our understanding of microbial communities and their intricate interactions. Various deep learning architectures, including convolutional neural networks, recurrent neural networks, autoencoders, and generative adversarial networks, have demonstrated promising results across several microbiome-related tasks. These tasks include taxonomic profiling, functional annotation, data augmentation, and disease prediction.

Despite the clear benefits of DL in managing high-dimensional, sparse, and complex microbiome data, several challenges remain. Issues such as overfitting, data leakage, interpretability, and data imbalance continue to hinder the robustness and generalizability of these models. Addressing these challenges will require the development of more sophisticated architectures, the application of regularization techniques, and the integration of explainable AI methods to enhance transparency and trust in model outcomes.

As the field of DL progresses, the importance of rigorous benchmarking for evaluating DL models becomes increasingly clear. Benchmarking is essential to ensure the reliability, reproducibility, and robustness of DL-based microbiome methods. Selecting evaluation metrics that align with the specific task, whether it be classification or regression, is critical to avoid biased reporting and to facilitate meaningful comparisons across different methods. Future advancements in microbiome DL research will hinge on addressing these benchmarking challenges. Developing standardized, community-driven benchmarks that take into account the unique characteristics of microbiome data will be crucial. Additionally, implementing transparent hyperparameter optimization practices and conducting unbiased comparative evaluations are essential for building trust in the results produced by new methods.

The manuscript primarily focuses on applications of deep learning for the analysis of amplicon and shotgun metagenomic data sets due to the wealth of research and advancements in this area. However, we also recognize the importance of exploring DL applications in other fields of microbiome research, such as metatranscriptomics, metabolomics, and proteomics, as well as studies focused on microbial interactions and dynamics beyond taxonomic and metabolic profiling.

As microbiome research generates increasingly large and intricate datasets, DL approaches are well-positioned to drive future innovations. Integrating multi-omics data, improving model interpretability, and developing novel architectures tailored to microbiome-specific tasks will be critical in unlocking deeper insights into microbial ecosystems. By overcoming current limitations, DL has the potential to revolutionize microbiome studies across medicine, agriculture, and environmental science, ultimately leading to new diagnostic, therapeutic, and ecological applications.

## Footnotes

^1^Homomorphic encryption is a form of cryptography that enables computations to be performed directly on encrypted data, eliminating the need for decryption.

^2^Cohen's d statistics is frequently used in estimating sample sizes for statistical testing.
