# Supplementary material for: Deep learning in microbiome analysis: a comprehensive review of neural network models
Source: Front Microbiol. 2025 Jan 22;15:1516667. doi: 10.3389/fmicb.2024.1516667 (PMC11794229; doi:10.3389/fmicb.2024.1516667)
Supplement: Supplementary file 1 [file Data_Sheet_1.pdf]

Supplementary 1 - Preliminaries of deep learning models

Supplementary Table 1. Essential Models with Suitable Input Data Types

Model	Data Structure	Model	Data Structure
FFNN (Feedforward Neural Network)	Vector	CNN (Convolutional Neural Network)	Images (3D Tensors)
RNN (Recurrent Neural Network)	Sequences	GNN (Graph Neural Network)	Graphs
LSTM (Long Short-Term Memory)	Sequences	GAN (Generative Adversarial Network)	Images / Tensors
GRU (Gated Recurrent Unit)	Sequences	Autoencoder	Vector / Images
MLP / MLPNN (Multilayer Perceptron Neural Network)	Vector	RBM (Restricted Boltzmann Machine)	Vector
SOM (Self-Organizing Map)	Vector	DRN (Deep Residual Network)	Images / Tensors
SVM (Support Vector Machine)	Vector	HN (Hopfield Network)	Vector
BM (Boltzmann Machine)	Vector	BiLSTM (Bidirectional LSTM)	Sequences
CGAN (Conditional GAN)	Images / Tensors	Transformer	Sequences

Supplementary table 2. Activation Functions in Microbiome Deep Learning Models

Activation Function	How it works	How to use	Formula	Python Implementation	Microbiome Task Examples
Sigmoid	Maps inputs to a range between 0 and 1, useful for binary classification.	Used in the final layer for binary output predictions or in hidden layers for simple transformations.	f(x) = 1 / (1 + e^-x)	`torch.nn.Sigmoid`, `tensorflow.keras.activations.sigmoid`	**Classification / Prediction**: Disease prediction (e.g., healthy vs diseased microbiome states).
ReLU (Rectified Linear Unit)	Outputs 0 for negative inputs and linear values for positive inputs, reducing vanishing gradient issues.	Commonly used in generator and discriminator models for non-linear transformations. Also, in convolutional layers to extract spatial features.	f(x) = max(0, x)	`torch.nn.ReLU`, `tensorflow.keras.activations.relu`	**Data Augmentation:** Generating synthetic microbiome data for model training. **Feature Extraction:** Detecting microbial patterns in metagenomic data.
Leaky ReLU	Similar to ReLU but allows a small gradient for negative inputs to avoid dead neurons.	Replaces ReLU when there are concerns about dying neurons in CNNs.	f(x) = x if x > 0, f(x) = αx otherwise (α is a small constant)	`torch.nn.LeakyReLU`, `tensorflow.keras.layers.LeakyReLU`	**Batch Correction:** Integrating datasets from various sources while mitigating batch effects.
Swish	Smooth activation function that improves optimization and accuracy in deep networks.	Used in hidden layers to enhance representation learning in complex data.	f(x) = x * sigmoid(x)	`tensorflow.keras.activations.swish`	**Data Imputation:** Filling in missing microbiome data points for longitudinal studies. **Clustering:** Grouping organisms or genes based on genetic similarity.
Tanh	Maps input to a range between -1 and 1, helpful for controlling	Used in the cell state and hidden state of LSTM to manage gradients.	f(x) = $\frac{e^{x}-e^{-x}}{e^{x}+e^{-x}}$	`torch.nn.Tanh`, `tensorflow.keras.activations.tanh`	**Microbiome interactions:** Longitudinal analysis of microbiome changes over time

	gradient flow in recurrent units.				
Softmax	Converts outputs into probabilities, with all outputs summing to 1.	Apply in the output layer for multiclass classification.	f(x_i) = $\frac{e^{x_i}}{\Sigma e^{x_j}}$	\`torch.nn.Softmax\`, \`tensorflow.keras.activations.softmax\`	**Classification / Prediction**: Prediction of more than 2 classes, e.g. predicting microbiome composition changes across sites or treatments.

Supplementary Table 3. Evaluation Metrics in Deep Learning

Metric	How to Interpret	Formula	Python Implementation
Accuracy	Measures the percentage of correctly classified samples.	$Accuracy = \frac{TP+TN}{TP+TN+FP+FN}$	\`sklearn.metrics.accuracy_score\`
Precision	Proportion of true positive predictions out of all positive predictions.	$Precision = \frac{TP}{TP+FP}$	\`sklearn.metrics.precision_score\`
Recall	Proportion of true positives detected out of actual positives.	$Recall = \frac{TP}{TP+FN}$	\`sklearn.metrics.recall_score\`
F1-Score	Harmonic mean of precision and recall.	$F_1 = \frac{2*Precision*Recall}{Precision+Recall}$	\`sklearn.metrics.f1_score\`
Mean Squared Error (MSE)	Measures the average squared difference between predicted and actual values.	$MSE = \frac{1}{n}\sum_{i=1}^{n}\left(actual_i - predicted_i\right)^2$	\`sklearn.metrics.mean_squared_error\`
Root Mean Squared Error (RMSE)	Square root of MSE; interprets error in the same units as the data.	$RMSE = \sqrt{MSE}$	\`numpy.sqrt\` for RMSE
Weighted Mean Absolute Percentage Error (WMAPE)	Measures prediction error as a percentage, weighted by the actual values.	$WMAPE = \frac{\sum_{i=1}^{n}\left(\lvert actual_i - predicted_i\rvert\right)}{\sum_{i=1}^{n}\left(\lvert actual_i\rvert\right)}$	Custom implementations using NumPy or pandas
Rate of Change of Feature Embeddings (RCFE)	Measures the rate at which embeddings change between iterations.	$RCFE = \sum_{t}\frac{\left(\lvert embedding_{t+1} - embedding_t\rvert\right)}{\lvert embedding_t\rvert}$	Custom implementations for feature dynamics analysis

Frechet Inception Distance (FID)	Evaluates similarity between generated and real data distributions.	$FID = \left\|\left\|\mu_1 - \mu_2\right\|\right\|^2 + Tr(\Sigma_1 + \Sigma_2 - 2\sqrt{\Sigma_1\Sigma_2})$	Custom GAN metrics libraries or PyTorch implementations
ROC-AUC	Area under the ROC curve; measures ability to distinguish between classes.	No single formula, based on the integration of the ROC curve.	`sklearn.metrics.roc_auc_score`
Log Loss	Evaluates incorrect confident predictions; lower values are better.	$Log\ Loss = -\sum_i (actual_i\ *\ log(predicted_i))$	`sklearn.metrics.log_loss`
Mean Absolute Error (MAE)	Measures the average absolute error between predictions and actuals.	$MAE = \frac{1}{n} \sum_i \|actual_i - predicted_i\|$	`sklearn.metrics.mean_absolute_error`
R-squared (R^2)	Indicates the proportion of variance explained by the model.	$R^2 = 1 - \frac{\Sigma_i(actual_i - predicted_i)^2}{\Sigma_i(actual_i - mean_i)^2}$	`sklearn.metrics.r2_score`
Reconstruction Loss	Measures how well the autoencoder can reconstruct the input data.	$ReconstructionLoss = \sum_i (input_i - output_i)^2$	`sklearn.metrics.mean_squared_error`
Cosine Similarity	Measures the cosine of the angle between two vectors to evaluate similarity.	$Cosine\ Similarity = \frac{dot(A, B)}{(\|\|A\|\| * \|\|B\|\|)}$	`sklearn.metrics.pairwise.cosine_similarity`

Supplementary Table 4. Risks of Applying Deep Learning in Microbiome Studies

Risk of Applying DL	Example from Microbiome	Solution
Overfitting due to small microbiome datasets	FFNN trained on a dataset predicting healthy vs diseased microbiome states produces high accuracy on training but fails on test data.	Use dropout, data augmentation, or cross-validation to reduce overfitting.
Interpretability issues in feature extraction	CNN identifies disease-associated microbial taxa but fails to explain why specific features were selected.	Integrate Explainable AI methods like SHAP or LIME to interpret feature contributions.
Data leakage during time-series analysis	Temporal microbiome data (e.g., predicting antibiotic effects on gut flora) leaks information from future to past sequences during training.	Ensure proper sequence partitioning and avoid overlap between training and validation sets.
Latent embeddings may obscure biological insights	Autoencoder compresses microbiome abundance data, but latent dimensions are not biologically interpretable.	Apply dimensionality reduction methods (e.g., t-SNE) to visualize latent space and use domain knowledge for interpretation.
Bias in synthetic data generation	GAN generates synthetic microbiome profiles but amplifies bias present in the original training data.	Use balanced training data and incorporate fairness-aware learning techniques.
Vanishing gradients in long sequences	RNN fails to capture long-term microbial interactions over extended time points.	Switch to LSTM or GRU (gated recurrent units) models to mitigate vanishing gradient issues.
Complexity in graph-based representations	Microbiome interaction networks represented as graphs lead to high computational costs and difficult model tuning.	Simplify graph structure using pruning techniques and optimize hyperparameters with grid or random search.

Supplementary Table 5. Comparative Analysis of Deep Learning Models in Microbiome Studies

Model	Strengths	Weaknesses	Connection to Other Models
Feedforward Neural Network (FFNN)	Simple and efficient for tabular data; good for small datasets.	Prone to overfitting; limited for sequential or spatial data.	Can be combined with Autoencoders for dimensionality reduction or feature extraction.
Recurrent Neural Networks (RNN)	Capture sequential data patterns; lightweight.	Suffer from vanishing gradients; limited for long sequences.	LSTM and GRU (gated recurrent units) improve upon traditional RNN weaknesses.
Long Short-Term Memory (LSTM)	Handle temporal dependencies; effective for time-series data.	Computationally intensive; struggles with very long sequences.	LSTM can extend RNN functionality, overcoming vanishing gradient problems.
Autoencoders	Great for unsupervised learning; reduce dimensionality.	Latent space can be biologically non-interpretable.	Often paired with FFNN or clustering models for downstream tasks.
Hopfield Networks	Good for pattern recognition and associative memory tasks.	Limited scalability to large datasets; less suited for deep architectures.	Can complement Boltzmann Machines for memory-based tasks.
Boltzmann Machines	Effective for unsupervised learning and probabilistic modeling.	High training complexity; requires careful tuning of hyperparameters.	Often used with Autoencoders for dimensionality reduction.
Convolutional Neural Network (CNN)	Extracts spatial or hierarchical features effectively; scalable.	Require large datasets; less interpretable.	Can be combined with RNN or LSTM for spatiotemporal analysis.
Generative Adversarial Networks (GAN)	Generate high-quality synthetic data; handles imbalanced datasets.	Difficult to train; risk of mode collapse.	GANs can leverage Autoencoders for realistic data generation.

Deep Residual Networks (DRNs)	Facilitate training of very deep networks; addresses vanishing gradient issues.	Prone to overfitting on small datasets; high computational cost.	ResNet can enhance feature extraction capabilities of CNNs.
Kohonen Networks	Effective for clustering and visualization tasks; unsupervised learning.	Limited to smaller datasets; lacks scalability for complex tasks.	Can work alongside Autoencoders for feature mapping.
Graph Neural Networks (GNN)	Handle relational data effectively; interpret complex graphs.	Computationally intensive; requires graph preprocessing.	Can integrate with CNN for spatial graph feature extraction.
Natural Language Processing (NLP)	Handle text and sequence data effectively; capture complex patterns.	Require large datasets for effective training; computationally expensive.	Attention mechanisms and transformers build on traditional NLP methods to enhance performance.
Attention and Transformers	Excel in capturing long-range dependencies and contextual patterns.	Require significant computational resources; complex architecture.	Often combined with RNNs for sequence-to-sequence tasks.

Literature recommendation:

For deep learning enthusiasts who are new to the field, several key resources offer both practical guidance and theoretical foundations. A book by François Chollet [3] is an excellent starting point for practitioners aiming to gain hands-on experience. Chollet introduces deep learning concepts through the Keras library, providing practical examples that simplify complex ideas. Additionally, the Keras documentation website serves as a valuable supplementary resource for implementation details and further learning.

For those seeking a deeper theoretical understanding, book [2] by Ian Goodfellow, Yoshua Bengio, and Aaron Courville offers a comprehensive exploration of the fundamental concepts and algorithms that are the basis of deep learning. This text is well-suited for readers interested in the mathematical and conceptual frameworks that drive the field. Complementing this, a book (and online resource) [1] by Aston Zhang, Zachary C. Lipton, Mu Li, and Alexander J. Smola provides an interactive approach that combines theoretical explanations with practical code examples, making it ideal for learners who appreciate a blend of theory and hands-on practice.

Additionally, a book [4] by Christopher M. Bishop and Hugh Bishop covers essential machine learning concepts that underpin deep learning techniques. While not exclusively focused on deep learning, this foundational text offers valuable insights into the mathematical frameworks and theoretical principles that are critical for understanding and advancing in the field.

References:

[1] Zhang, A., Lipton, Z. C., Li, M., & Smola, A. J. (2023). Dive into Deep Learning. Cambridge University Press.

[2] Goodfellow, I., Bengio, Y., & Courville, A. (2017). Deep Learning (Vol. 1). Cambridge, MA, USA: MIT Press.

[3] Chollet, F. (2021). Deep Learning with Python. Simon and Schuster.

[4] Bishop, C. M., & Bishop, H. (2023). Deep Learning: Foundations and Concepts. Springer Nature.
