# Supplementary material for: Deep learning in microbiome analysis: a comprehensive review of neural network models
Source: Front Microbiol. 2025 Jan 22;15:1516667. doi: 10.3389/fmicb.2024.1516667 (PMC11794229; doi:10.3389/fmicb.2024.1516667)
Supplement: Supplementary file 2 [file Data_Sheet_2.pdf]

Supplementary 2 - Selected models

1. VAMB (Variational Autoencoders for Metagenomic Binning)

Model

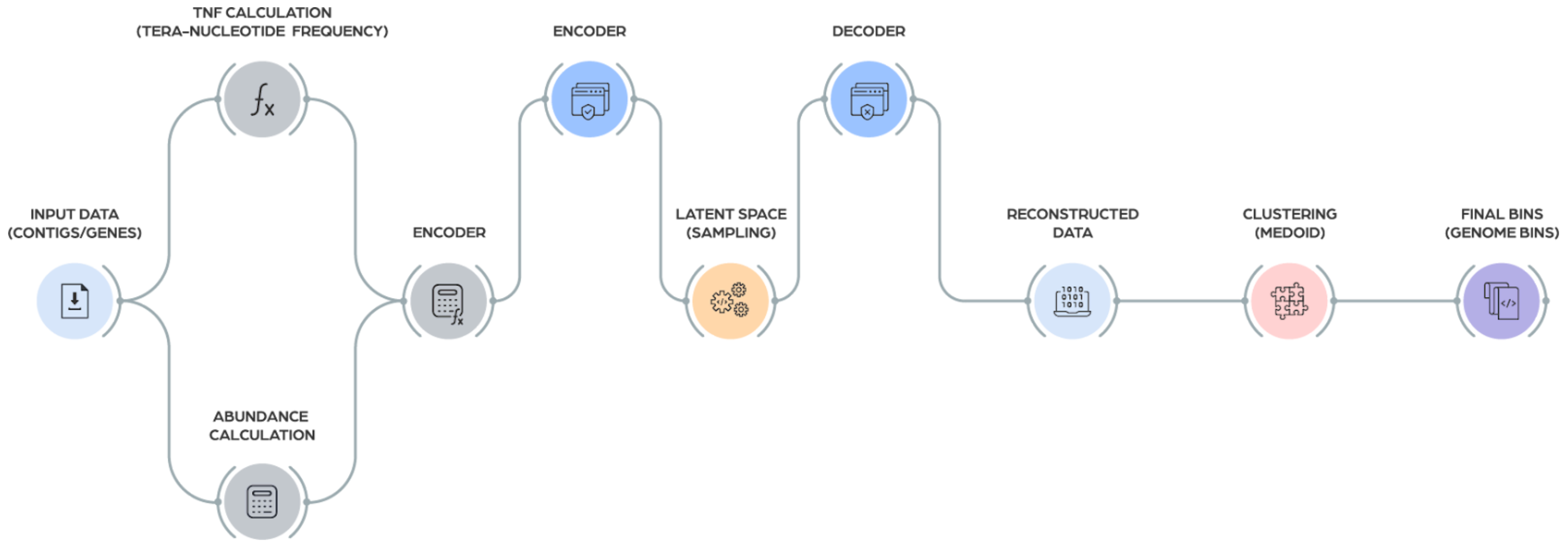

Paper

Nissen JN, Johansen J, Allesøe RL, Sønderby CK, Armenteros JJA, Grønbech CH, et al. Improved metagenome binning and assembly using deep variational autoencoders. Nat Biotechnol. mayo de 2021;39(5):555-60.

Description

VAMB is a deep learning-based algorithm that utilizes Variational Autoencoders (VAE) for binning metagenomic sequences by integrating co-abundance and compositional data.

Problem Solved

Improving the accuracy and completeness of metagenomic binning by better clustering genomic sequences from different samples.

Input Data

- **Metagenomic Sequences**: Raw sequences from metagenomic samples.
- **Abundance Data**: Information on the abundance of sequences across different samples.

Architecture Steps

1. **Compute TNF and Abundance**:
    - **Tetranucleotide Frequencies (TNF)**: Calculation of 4-mer frequencies from the sequences.
    - **Abundances**: Estimation of sequence abundances based on read mappings.
2. **Concatenate TNF and Abundance**: Combining TNF and abundance data into a single input matrix.
3. **VAE Training**: Training the VAE to encode the concatenated data into a latent space.
4. **Latent Representation**: Obtaining the compressed representation of the data.
5. **Medoid Clustering**: Grouping the latent representations using an iterative medoid clustering algorithm.
6. **Generating Genome Bins**: Final grouping of sequences into genomic bins.

Output Data

- **Genome Bins**: Accurately grouped genomic sequences.

2. PhaMer (Accurate Identification of Bacteriophages from Metagenomic Data using Transformer)

Model

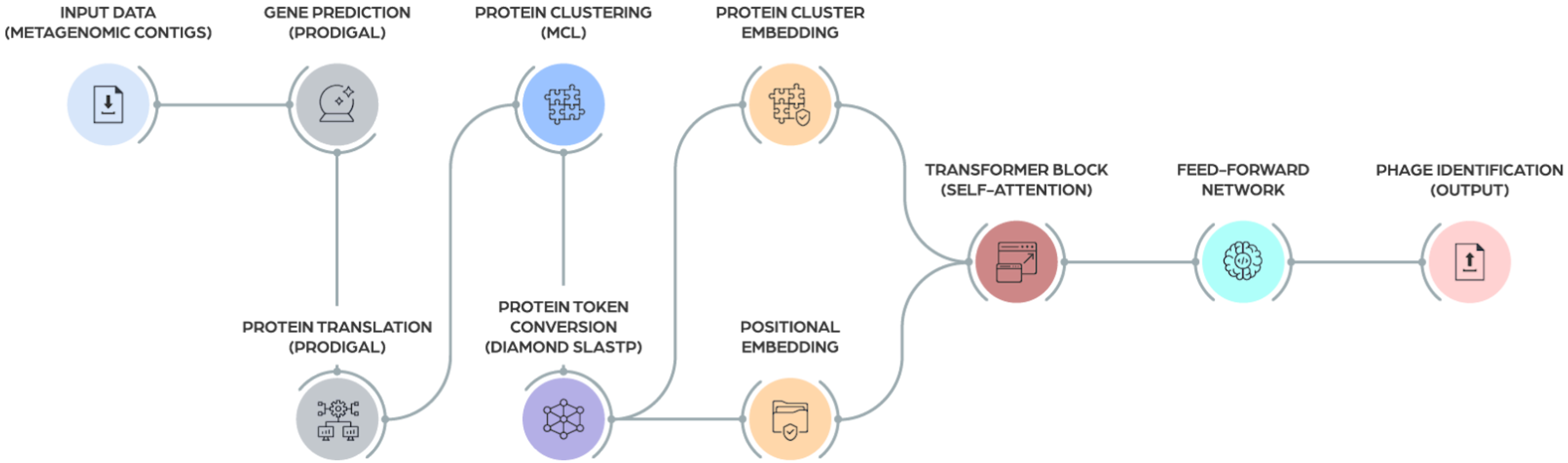

Paper

Shang, J., Tang, X., Guo, R., & Sun, Y. (2022). Accurate identification of bacteriophages from metagenomic data using Transformer. Briefings in Bioinformatics, 23(4), bbac258.

Description

PhaMer is a bioinformatics tool that utilizes a Transformer-based model for the accurate identification of bacteriophages from metagenomic data.

Problem Solved

Accurate identification of bacteriophages in metagenomic data, which is crucial for understanding microbiome dynamics and host interactions.

Input Data

- **Metagenomic Contigs**: Raw DNA sequences from metagenomic samples.
- **Pfam Annotations**: Information on protein families obtained using HMMER and Pfam.

Architecture Steps

1. **Gene Prediction (Prodigal)**: Identifying open reading frames (ORFs) in contigs.
2. **Protein Translation (Prodigal)**: Translating ORFs into protein sequences.
3. **Protein Clustering (MCL)**: Grouping similar proteins using the Markov Clustering Algorithm.
4. **Protein Token Conversion (DIAMOND BLASTP)**: Converting protein sequences into protein cluster tokens.
5. **Protein Cluster and Positional Embedding**: Representing protein clusters and their positions as numerical vectors.
6. **Transformer Block (Self-Attention)**: Learning dependencies between protein cluster tokens.
7. **Feed-Forward Network**: Processing the output from the Transformer block for prediction.
8. **BGC Class Prediction**: Classifying contigs as bacteriophages or non-phages.

Output Data

- **Predicted BGC Classes**: Probability assessment indicating whether a contig is a bacteriophage.

3. Multi-Layer and Recursive Neural Networks for Metagenomic Classification

Model

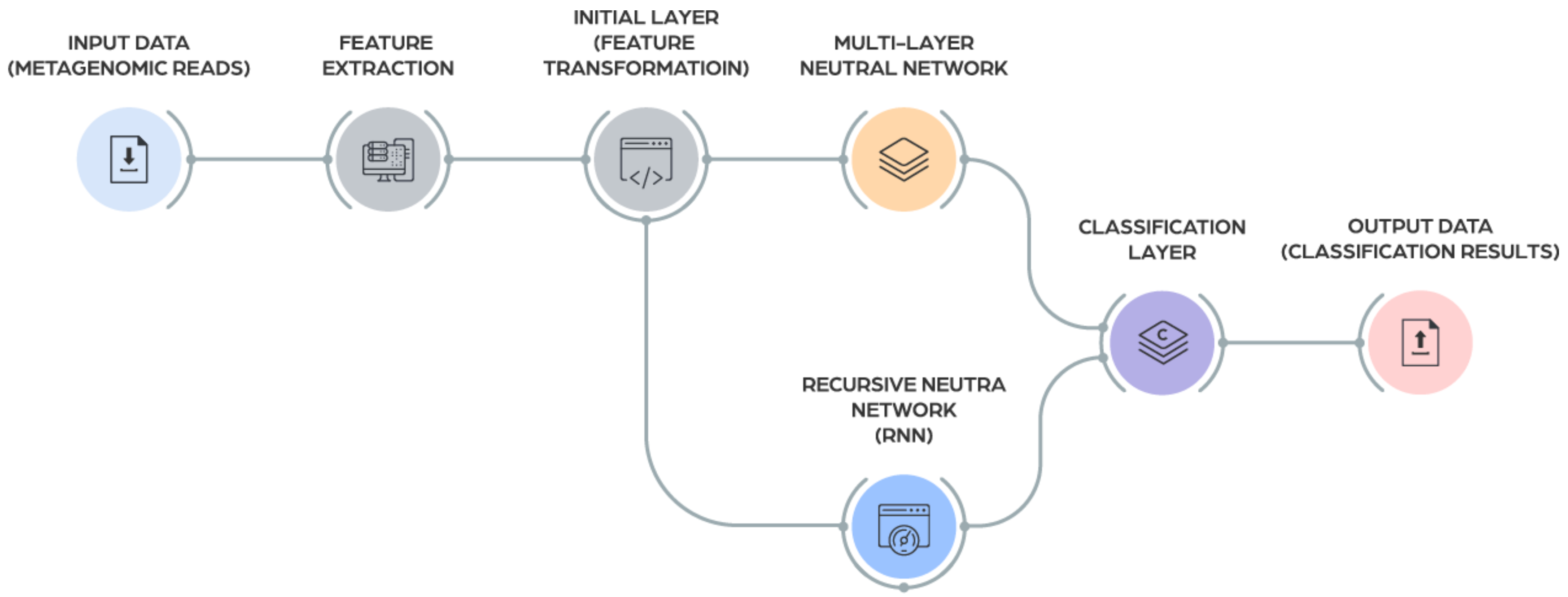

Paper

Ditzler G, Polikar R, Rosen G. Multi-Layer and Recursive Neural Networks for Metagenomic Classification. IEEE Trans Nanobioscience. septiembre de 2015;14(6):608-16.

Description

The study by Ditzler et al. presents the application of multi-layer and recursive neural networks for the classification of metagenomic data.

Problem Solved

Improving the accuracy and efficiency of metagenomic data classification through advanced deep learning techniques.

Input Data

- **Metagenomic Reads**: Raw DNA sequences from various microorganisms.

Architecture Steps

1. **Feature Extraction**: Transforming raw reads into numerical representations (e.g., k-mer frequency counts).
2. **Initial Layer (Feature Transformation)**: Normalization or other preprocessing steps to prepare data for neural network processing.
3. **Multi-Layer Neural Network**: Deep neural network layers for extracting high-level features.
4. **Recursive Neural Network (RNN)**: Capturing sequential dependencies and patterns in the reads.
5. **Classification Layer**: Final layer responsible for classification.
6. **Classification Results**: Assigning reads to specific categories (e.g., bacterial species, functional genes).

Output Data

- **Classification Results**: Categories assigned to metagenomic reads.

4. Deep Restricted Boltzmann Machines for Causal Inference

Model

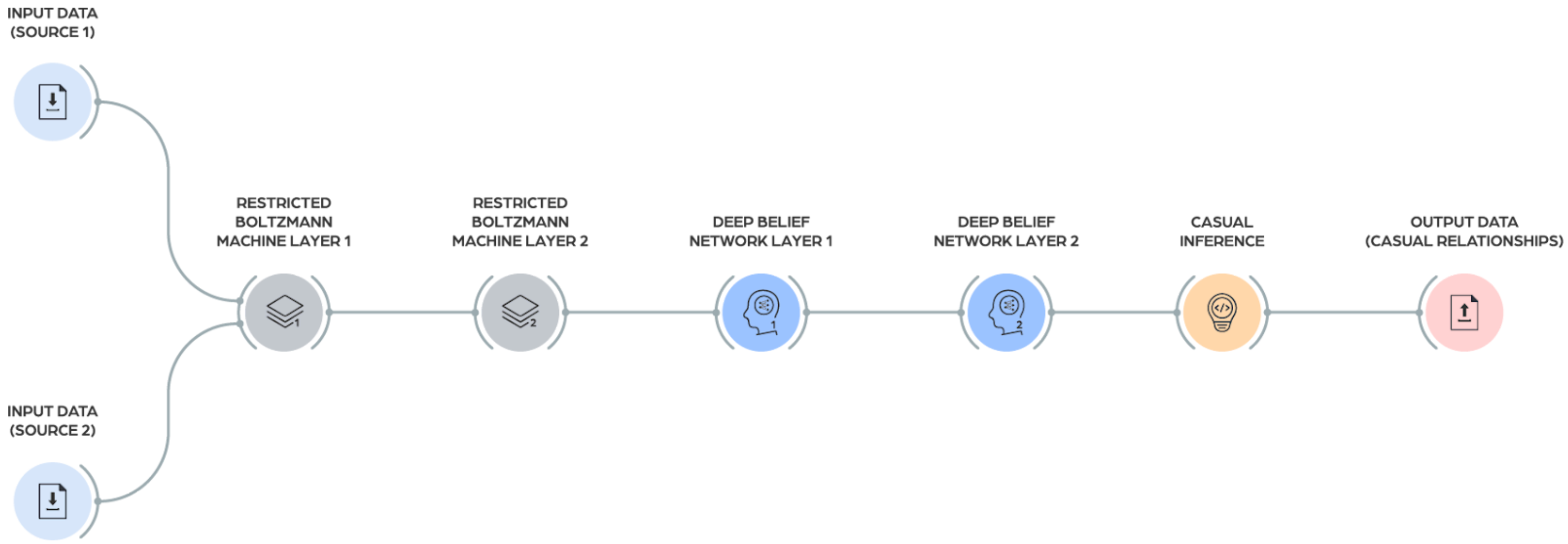

Paper

Sokolovska N, Clément K, Zucker JD. Revealing causality between heterogeneous data sources with deep restricted Boltzmann machines. Inf Fusion. 1 de octubre de 2019;50:139-47.

Description

The study by Sokolovska et al. presents the use of Deep Restricted Boltzmann Machines (RBM) for uncovering causality between heterogeneous data sources.

Problem Solved

Discovering causal relationships between different data sources by modeling complex interactions using deep neural networks.

Input Data

- **Heterogeneous Data**: Data from various sources, which can include images, text, numerical data, etc.

Architecture Steps

1. **Restricted Boltzmann Machine (RBM) Layers**: Processing input data through successive RBM layers to learn patterns and correlations.
2. **Deep Belief Network (DBN) Layers**: Utilizing Deep Belief Networks (DBN) to further extract abstract features from RBM outputs.
3. **Causal Inference**: Analyzing the extracted features to infer causal relationships between different data sources.
4. **Generating Causal Relationships**: Determining potential causal links based on the model.

Output Data

- **Causal Relationships**: Discovered causal links between different data sources.

5. TaxoNN (Ensemble of Neural Networks on Stratified Microbiome Data for Disease Prediction)

Model

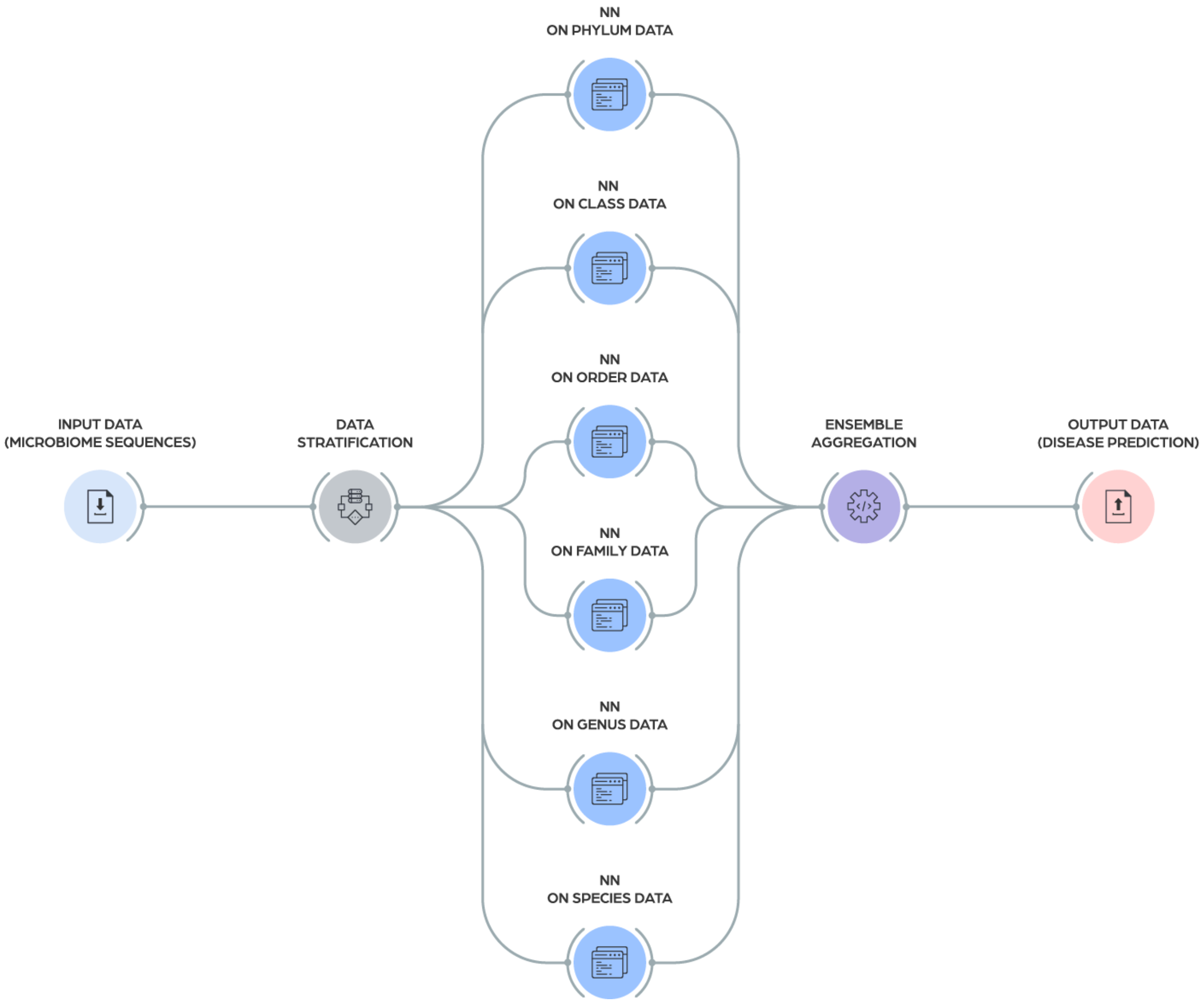

Paper

Sharma D, Paterson AD, Xu W. TaxoNN: ensemble of neural networks on stratified microbiome data for disease prediction. Bioinforma Oxf Engl. 1 de noviembre de 2020;36(17):4544-50.

Description

TaxoNN is an ensemble model that utilizes a set of neural networks trained on stratified microbiome data at different taxonomic levels for disease prediction.

Problem Solved

Enhancing disease prediction accuracy by employing a multi-layered approach to analyze microbiome data across various taxonomic levels.

Input Data

- **Microbiome Sequences**: Raw DNA sequences from microbiome samples.

Architecture Steps

1. **Data Stratification**: Dividing microbiome data into different taxonomic levels (e.g., phylum, class, order, family, genus, species).
2. **Neural Networks for Each Level**: Training separate neural networks on data from each taxonomic level.
3. **Ensemble Aggregation**: Combining the outputs of these neural networks to form a final prediction.
4. **Disease Prediction**: Generating a disease prediction based on the aggregated results from the ensemble of neural networks.

Output Data

- **Disease Prediction**: Final disease prediction based on aggregated results from neural networks across different taxonomic levels.
